# *Legionella pneumophila* cell surface RtxA release by LapD/LapG and its role in virulence

**DOI:** 10.1186/s12866-024-03395-1

**Published:** 2024-07-19

**Authors:** Hussein Kanaan, Annelise Chapalain, Ali Chokr, Patricia Doublet, Christophe Gilbert

**Affiliations:** 1 Centre International de Recherche en Infectiologie (CIRI), Université Lyon 1, INSERM U1111, CNRS UMR5308, ENS, Lyon Bât. Rosalind Franklin, 50 avenue Tony Garnier, Lyon, 69007 France; 2 Research Laboratory of Microbiology (RLM), Department of Life and Earth Sciences, Faculty of Sciences I, Lebanese University, Hadat Campus, Beirut, Lebanon

**Keywords:** *L. Pneumophila*, RTX family, LapD, LapG, Type 1 secretion system, Virulence

## Abstract

**Background:**

*Legionella pneumophila* is a Gram-negative intracellular bacillus and is the causative agent of a severe form of pneumonia called Legionnaires’ disease which accounts for 2-9% of cases of community acquired pneumonia. It produces an extremely large protein belonging to the RTX (*R*epeats in *T*o*X*in) family, called RtxA, and we previously reported that RtxA is transported by a dedicated type 1 secretion system (T1SS) to the cell surface. RTX proteins have been shown to participate in the virulence or biofilm formation of various bacteria, the most studied models being the pore forming hemolysin A (HlyA) of *Escherichia coli* and the biofilm associated protein LapA of *P. fluorescens.* LapA localization depends on the enzymatic release by LapD/LapG complex activity. This study aimed to elucidate the dual localization (cell surface associated or released state) of *L. pneumophila* RTX protein (RtxA) and whether this released versus sequestered state of RtxA plays a role in *L. pneumophila* virulence.

**Results:**

The hereby work reveals that, in vitro, LapG periplasmic protease cleaves RtxA N-terminus in the middle of a di-alanine motif (position 108–109). Consistently, a strain lacking LapG protease maintains RtxA on the cell surface, whereas a strain lacking the c-di-GMP receptor LapD does not exhibit cell surface RtxA because of its continuous cleavage and release, as in the LapA-D-G model of *Pseudomonas fluorescens*. Interestingly, our data point out a key role of RtxA in enhancing the infection process of amoeba cells, regardless of its location (embedded or released); therefore, this may be the result of a secondary role of this surface protein.

**Conclusions:**

This is the first experimental identification of the cleavage site within the RTX protein family. The primary role of RtxA in *Legionella* is still questionable as in many other bacterial species, hence it sounds reasonable to propose a major function in biofilm formation, promoting cell aggregation when RtxA is embedded in the outer membrane and facilitating biofilm dispersion in case of RtxA release. The role of RtxA in enhancing the infection process may be a result of its action on host cells (i.e., PDI interaction or pore-formation), and independently of its status (embedded or released).

**Supplementary Information:**

The online version contains supplementary material available at 10.1186/s12866-024-03395-1.

## Background

The *Legionella* genus comprises around 60 species including 70 serogroups. Thirty of these species are pathogenic to humans, especially *Legionella pneumophila* serogroup 1 (Lp1) that is responsible for more than 90% of legionellosis incidents [1–3]. *L. pneumophila* is a Gram-negative intracellular bacillus and is the causative agent of a severe form of pneumonia called Legionnaires’ disease which accounts for 2-9% of cases of community acquired pneumonia [1, 2, 4]. These bacteria inhabit fresh water environments as parasites of protozoa, especially amoebae, which are considered their natural hosts [1]. However, humans are considered accidental hosts to *L. pneumophila*. In fact, legionellosis is almost exclusively caused by *Legionella* contaminations originating from man-made environments such as water-cooling towers, hot and cold water systems and spa pools where bacteria use dispersed aerosols to infect human alveolar macrophages [1, 5]. Nosocomial instances of legionellosis have also been frequently recorded due to the presence of *Legionella* in hospitals’ water supply despite appropriate maintenance of water distribution systems [6]. Overall, the mortality rate of Legionnaires’ disease ranges from 7 to 25% despite appropriate antibiotic treatment which renders this disease a public health concern [7].

In the case of pathogenic bacteria, secretion systems constitute a crucial factor for pathogenesis and interbacterial competition mainly through damaging and/or manipulating the host, evading the immune system and establishing a replicative niche in case of intracellular pathogens such as *L. pneumophila* [8, 9]. Regarding *Legionella*, one study reports the presence of T1SS, T2SS, T4SS, and a T6SS; this was previously unknown [10]. T2SS and T4SS were directly correlated with *L. pneumophila* virulence, particularly T4SS which allows this bacterium to assume an intracellular lifestyle [11]. Regarding the T1SS, we previously reported that it is responsible in *L. pneumophila* for the secretion of a large RTX protein called RtxA [12]. Typically, T1SSs consist of three components: an inner membrane ABC transporter that relies on ATP for substrate transport, a periplasmic membrane fusion protein and an outer membrane protein; in *L. pneumophila* these are named LssB, LssD and TolC respectively [12]. These proteins form a channel spanning both bacterial membranes for substrate secretion. Usually, this secretion system transports its substrate in a single step from the bacterial cytoplasm to the extracellular medium. However, recent studies suggest that some RTX transporting T1SSs mediate transport via a two-step process that involves a periplasmic intermediate [13, 14].

**Fig. 1:**
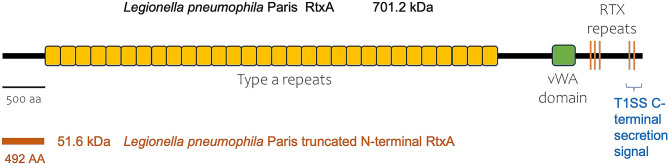
Schematic representation of the *L. pneumophila* RtxA protein. The *L. pneumophila* Paris RtxA (701.2 kDa; top of the figure) contains the traditional components of an RTX protein such as type a repeats (30 in *L. pneumophila* Paris) in addition to a Von Willebrand factor type A domain (*vWA*) and C-terminal RTX repeats. The T1SS secretion signal is located at the COOH-end of RtxA. The truncated N-terminal RtxA domain (51.6 kDa; bottom of the figure) was used in the hydrolysis assay with LapG corresponded to the first (N-terminal) 492 AAs

RTX proteins have been shown to participate in the virulence or biofilm formation of various bacteria, the most studied models being the pore forming hemolysin A (HlyA) of *Escherichia coli* [15, 16] and the biofilm associated protein LapA of *P. fluorescens* [14, 17]. Regarding the adhesin LapA of *P. fluorescens*, studies report that biofilm formation in this bacterium is dependent on the presence of LapA on the cell surface and that specific environmental signals orchestrate this process [18]. Briefly, the inner membrane effector protein LapD (member of GGDEF/EAL domain proteins) in conjunction with a diguanylate cyclase [19] senses changes in the intracellular cyclic-di-GMP levels and regulates accordingly, the activity of a periplasmic protease LapG by sequestering or releasing it in the periplasm [20]. This protease, when free, can cleave the N-terminus of LapA leading to its release and biofilm dispersal and vice versa [21]. The *L. pneumophila* RtxA shares the general features of RTX proteins such as a C-terminal secretion signal, an RTX repeats motif (GGXGXDX) and a central repeat region [22, 23]. More specifically, the Paris strain used in this study exhibits 30 type a repeats with similarity to the “Thrombospondin type 3 repeat” of human endothelial cells (Fig. 1) [22]. This domain was shown to bind fibrinogen, fibronectin, laminin and collagens [24]. Additional domains can be detected such as von Willebrand factor type 1 (vWA) and several blocks of tandem RTX repeats [22]. The vWA domain is involved in adhesion processes, whereas the RTX repeats are related to adhesion and cytotoxicity probably by pore formation. According to the LapA model in *Pseudomonas* [13], RtxA is proposed to be secreted in a C-terminal to N-terminal direction (T1SS), but secretion of RtxA is stalled during translocation, leaving it threaded through TolC (the outer membrane component) with its cleavable N-terminal retention domain localized in the periplasm and multiple adhesive repeats exposed at the cell surface. Thus, RtxA exposure on the cell surface is proposed to be the results of this blockage inside the TolC canal. Concerning its possible role in virulence, Δ*rtxA* mutants exhibited a measurable loss of virulence and reduction in adhesion capabilities to monocytic and epithelial cells [25]. RtxA would also be involved in intracellular survival such as inhibition of lysosomal fusion, but this role has not been firmly established [26]. In addition, functional LapG and LapD homologs were identified in *L. pneumophila*, but their role with T1SS was not studied [27, 28].

To date, it is not known whether *L*. *pneumophila* RtxA is regulated on the cell surface in a manner similar to other closely related adhesins. In this paper, we provide evidence that RtxA^Nterm^ is cleaved by LapG in vitro. We prove that this cleavage ultimately results in the release of RtxA from the cell surface in vivo. Interestingly, it seems that the T1SS and LapD/LapG co-evolved in *L. pneumophila* species and harbored specific characteristics compared to the equivalent systems present in other *Legionella* species. Furthermore, we investigated the role of RtxA and its Type 1 secretion system in *L. pneumophila* virulence. By using various mutants and specific neutralizing RtxA antibodies, we showed the enhancing role of RtxA in the early steps of *Legionella* infection regardless of its location (embedded in the cell outer membrane or released into the environment).

## Results

### In-vitro cleavage of RtxA by LapG

Previous studies report that some RTX family proteins are susceptible to cleavage by periplasmic proteases in a controlled regulatory process [18, 21]. And since homologs of both substrate and enzyme were identified in *L. pneumophila* [27], we hypothesized that the periplasmic protease LapG can indeed cleave RtxA. Given that the size of the entire RtxA protein in *Legionella pneumophila* Paris strain is 6765 amino acids [22], a truncated COOH-His-tagged fragment of RtxA^Nterm^ (492 amino acids ~ 51.6 kDa; Fig. 1), and a NH_2_-His-tagged LapG protein minus its secretion signal (188 amino acids ~ 22 kDa) were cloned in appropriate plasmids and overproduced in *E. coli*. Both expression in *E. coli* and subsequent purification were conducted independently.

**Fig. 2:**
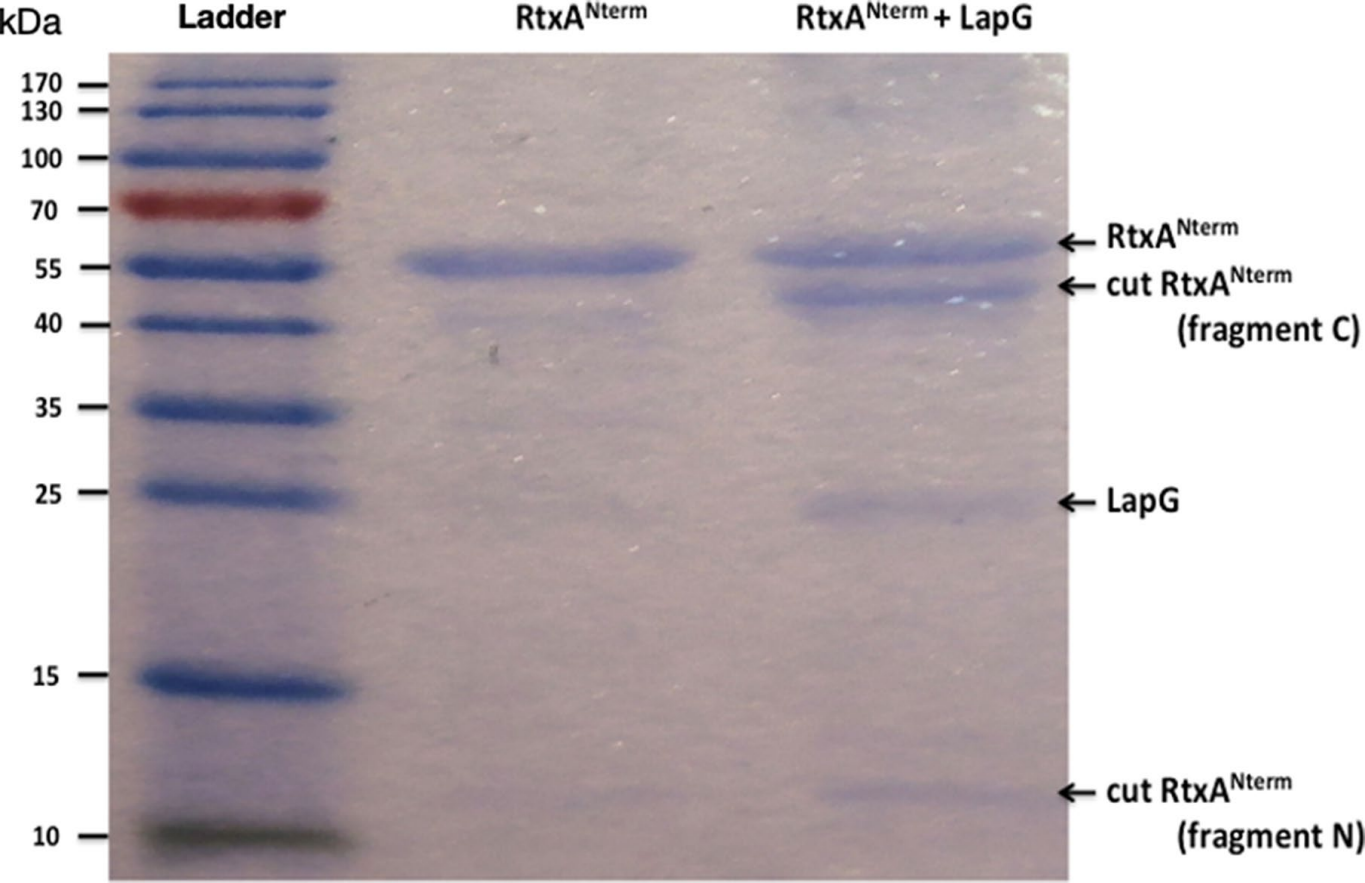
Gel electrophoresis of RtxA^Nterm^ incubated with LapG protease. Purified RtxA^Nterm^ (shown in the second lane ~ 55 kDa) was incubated with LapG (~ 23 kDa) in an elution buffer from TAKARA purification kit (50 mM sodium phosphate, 300 mM NaCl, 150 mM imidazole, pH 7.4) containing 40 mM MgCl_2_ and 80 mM CaCl_2_ for 2 h at 37 °C. The third lane represents the outcome of the incubation showing two protein bands at 42 and 12 kDa corresponding to the COOH and NH_2_ fragments of the cleaved RtxA^Nterm^ in addition to residual uncleaved amount of the latter protein and LapG. Proteins were run on a 12% polyacrylamide gel, PageRuler™ pre-stained protein ladder is used

Following co-incubation of the two purified proteins in a suitable buffer for 2 h, reaction mixture components were visualized using SDS-PAGE (Fig. 2; supporting information Figure S8).

In the control lane corresponding to RtxA^Nterm^ incubated alone in the assay buffer, no degradation was observed during the 2 h incubation period. On the contrary, the lane containing the co-incubated proteins show two protein bands (42 and 12 kDa) in addition to LapG and RtxA^Nterm^ which indicates that part of RtxA^Nterm^ was cleaved in vitro. Furthermore, the sum of the molecular weights of the 2 RtxA^Nterm^ post-cleavage fragments is consistent with that of the full-length recombinant protein (54 kDa).

**Fig. 3:**
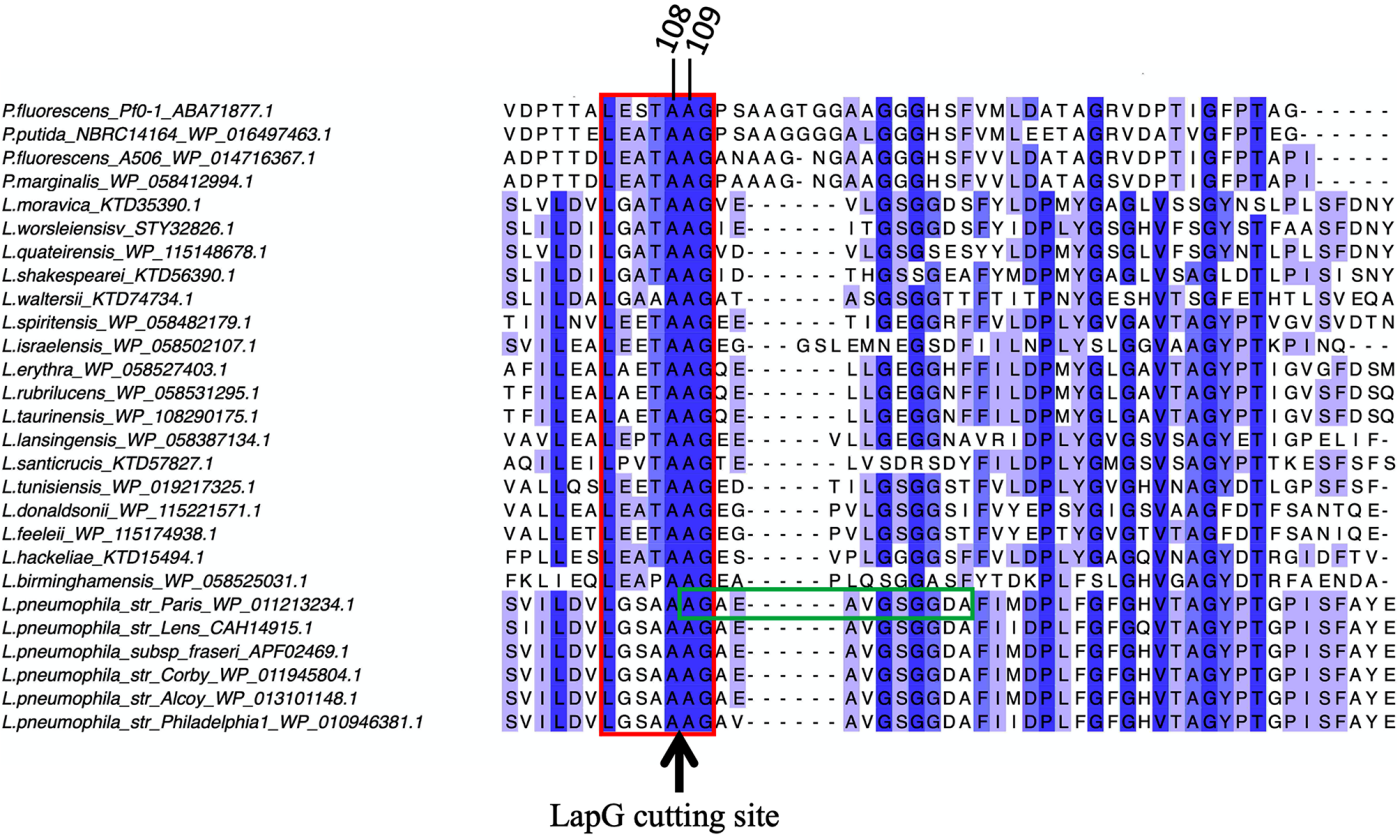
Primary sequence alignment of LapG cleavage site region of various potential predicted RtxA proteins within *Legionella* species with *Pseudomonas* LapA proteins. Cleavage site between two alanine residues is indicated by an arrow. The dashes correspond to gaps in aminoacid sequences. The red box surrounds a highly conserved region close to the cleavage site recognized by the LapG protease. The green box surrounded sequence is the outcome of Edman degradation analysis of the cleaved RtxA C-fragment, with cleavage occurring between residues at positions 108 and 109, shown at the top of the figure. This position is well conserved in most of RtxA and LapA proteins with few variations (from 106 to 112 for the first alanine). Purple shading gradation highlights amino acid conservation from an intense purple color (indicating highly conserved residues) to a light purple color (indicating poor conservation). Alignment of RtxA cutting site regions was performed with Jalview software using Clustal Omega software to align the protein sequences (version 2.10.5; [29])

These fragments also coincided with previous reports stating the most probable cleavage site to be around 108th or 109th residue. Therefore, to further pinpoint the exact in vitro cleavage site, the C-fragment (42 kDa) of the cleaved RtxA^Nterm^ was sequenced by Edman degradation and the identity of its 7 N-terminal amino acids was found to be AGAEAVG, indicating that the cleavage occurs between residues 108 and 109. This result experimentally confirmed that the *L. pneumophila* RtxA cleavage site is consistent with the putative cleavage site proposed for other RTX proteins, like the one of *P. fluorescens* [18] (Fig. 3).

Interestingly, using protein BLAST search, RtxA N-terminus homologues were identified among many *Legionella* species and the alignment of LapG cutting site region revealed few differences. Most notably the threonine residue in position 107 (before the double A recognized cutting site), present in most cleavage sites among LapA family proteins and *Legionella* species (except *L. waltersii* and *L. birmighamensis*), is substituted by an alanine residue in all *L. pneumophila* RtxA proteins (Fig. 3). Together, these data emphasize that *L. pneumophila* RTX proteins defines a well conserved sub-clade within the closely related RTX proteins in the *Legionella* clade but distinct from *Pseudomonas* LapA. The phylogeny of the first N-terminal 210 amino acids from predicted proteins supports this observation (supporting information Figure S1).

### Phylogeny of LapD/LapG system among *Legionella* species

Recently, T1SS was reported to be restricted to *L. pneumophila* species among the genus *Legionella* [10]. However, the authors searched for the entire *lss* operon in *Legionella*, but the whole genetic organization does not seem to be conserved among the genus which may have led the authors to such conclusion. Conversely, our BLAST search found RtxA homologs in many *Legionella* species, which corroborates the results of another recent study reporting the presence of C39-like T1SS exporters involved in “adhesin-like” export (T1SS-RtxA system) in 19 *Legionella* species among 45 *Legionella* species studied [30].

**Fig. 4:**
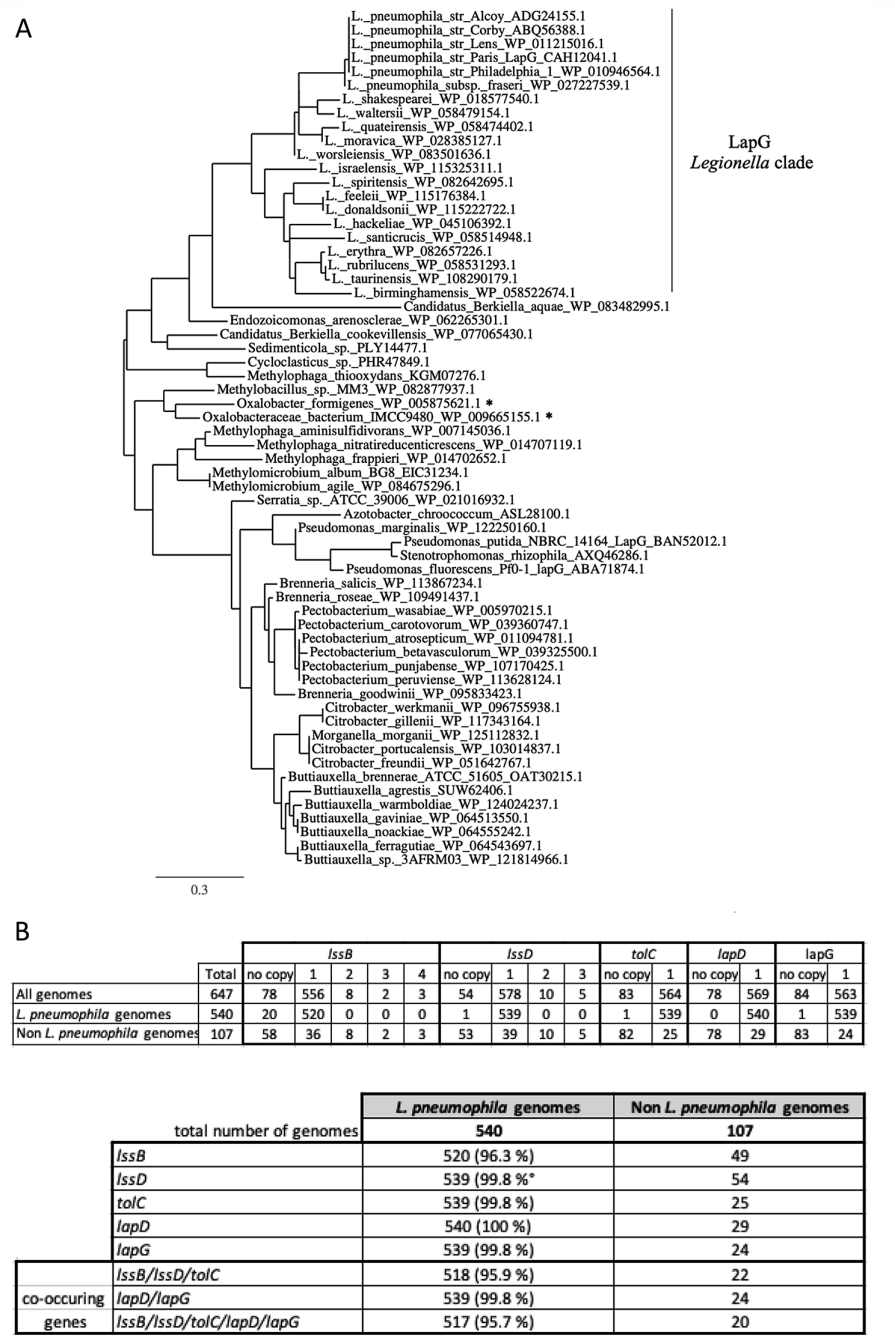
LapG family proteins phylogenetic tree inferred using maximum-likelihood (PhyML 3.0 software). **(A)** The proteins reported in the tree were chosen for their high similarities with *Pseudomonas* LapG. Only one representative protein is conserved in each bacterial species except in *Legionella pneumophila*. All bacterial species are Gammaproteobacteria except the two marked with an asterisk which are in Betaproteobacteria class. The branch length is proportional to the number of substitutions per site (scale at the bottom). L: *Legionella.* Phylogenetic trees were inferred using maximum-likelihood with PhyML 3.0 software online pipeline (http://phylogeny.lirmm.fr; [31]). **(B)** Distribution of the genes *lssB*, *lssD*, *tolC*, *lapD* and *lapG* (with gene copy number) from *Legionella* in 647 different *Legionella* strains including *L. pneumophila* and *non-pneumophila* strains. The protein sequences of interest of *L. pneumophila* strain were blasted against our genome database of family-clustered proteins to determine the presence or absence of proteins in the corresponding genomes [32]

Thus, we performed protein BLAST searches on published *Legionella* genomes looking for LapG and LapD proteins and components of RtxA/LapA cleavage system, in order to construct phylogenetic trees (LapG phylogeny shown on Fig. 4A).

Interestingly, we were able to identify LapD and LapG in many species of the *Legionella* genus that form a clade within *L. pneumophila* strains. A similar tree was obtained using LapD homologous proteins (supporting information Figure S2).

To further analyze the presence of T1SS genes and *lapD*/*lapG* genes in *Legionella* genomes, we studied their distribution in 647 *Legionella* genomes (including 540 *Legionella pneumophila* strains; Fig. 4B). As the *rtxA* gene is a very long gene harboring many repeat sequences, it is often not annotated in genomes and sequencing data might correspond to uncertain sequence area for these genomes. Thus, we did not include *rtxA* genes in the analysis at this stage. In a few cases, *lssB* or *lssD* genes were present in more than one copy, but only within non-*pneumophila* genomes. Interestingly, studying the co-occurrence of T1SS genes, *lapD*/*lapG* and all the five genes together revealed high conservation of the global machinery (secretion and release of RtxA) among *Legionella pneumophila* species (over 95% co-occurrence; Fig. 4B). It is worth to note that these genes are located in two different loci in *Legionella pneumophila* Paris genome: a first one with *lssB* and *lssD*, a second one with *tolC, lapD* and *lapG*, whereas *rtxA* is present in a third independent locus.

### RtxA release from cell surface is controlled by LapD/LapG system in vivo

We previously demonstrated that *L. pneumophila* RtxA, as other RTX proteins is transported by a T1SS, namely the LssB/LssD /TolC T1SS [12]. However, the possible fates of the protein after its passage through the secretion machinery were not clear. Following evidence from *P. fluorescens* [21] and building on the results of our previous in vitro cleavage assays, we hypothesized that LapG cleavage of RtxA results in its release from the cell surface of *Legionella*.

Specific rabbit polyclonal antibodies against the C terminal region of RtxA were produced. *L. pneumophila* Δ*rtxA*, Δ*lapG* and Δ*lapD* deletion mutants were designed and constructed to abolish the RtxA production or to disrupt the hypothetical RtxA release process. The Δ*rtxA* mutant strain was used as a negative control in immunofluorescence microscopy.

**Fig. 5:**
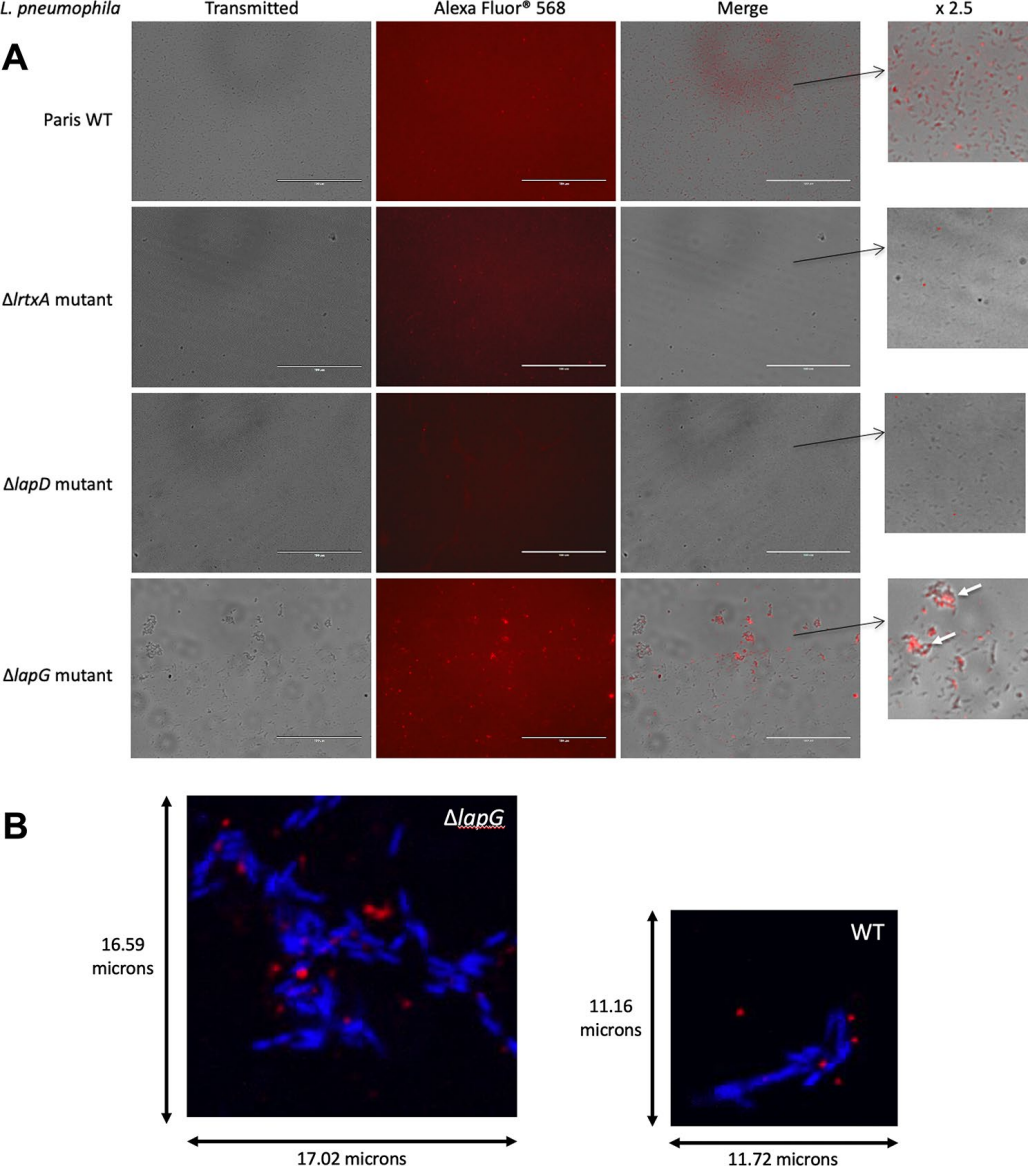
Immunofluorescence microscopy of four *L. pneumophila* strains using anti RtxA^Cterm^ antibodies. **(A)** Three days old cultures of *L. pneumophila* Paris wild type, Δ*lapG* mutant, Δ*lapD* mutant and Δ*rtxA* mutant were used in an indirect immunofluorescence assay using antibodies targeting the C-terminus of RtxA protein at 0.374 µg ml^-1^. The secondary antibody used was Alexa Fluor^®^ 568 goat anti-rabbit (Invitrogen Inc. USA). Scale bar shown is 100 μm. Aggregated bacteria are pointed out with white arrows. **(B)** Confocal microscopy image of *L. pneumophila* Paris and ∆*lapG* strains stained with DAPI and immunolabelled using antibodies targeting the C-terminus of RtxA protein at 0.374 µg ml^-1^. The secondary antibody used was Alexa Fluor^®^ 594 goat anti-rabbit (Invitrogen Inc. USA)

As expected, wild type cells (WT, Fig. 5A) show clear fluorescence dots on the contrary to Δ*rtxA* mutants which are defective for RtxA production and hence do not exhibit any fluorescence.

Few red dots are present on the image but that might result from antibody aggregates with no link to bacteria cells. Therefore, our negative control is efficient and no cross-reaction of our primary antibodies with other *Legionella* proteins is detected. In addition, as almost all WT bacteria were marked with red fluorescent dots, our experimental growth conditions seemed to maintain a significant amount of *L. pneumophila* RtxA on cell surface. Concerning the Δ*lapD* strain, the absence of fluorescence associated with bacteria cells allowed us to conclude that no RtxA was present at the surface of the cells (Fig. 5A). This result is consistent with the proposed role of LapD. Indeed, our results show that LapD controls the activity of the periplasmic protease LapG, possibly by physically sequestering it close to the outer-face of the inner membrane (similarly to *P. fluorescens* LapA [27]) and preventing RtxA N-terminus periplasmic cleavage. Therefore, the absence of LapD in the ∆*lapD* mutant strain may result in continuously free LapG in the periplasm that is able to cleave and release RtxA from the cell surface. Finally, the strain lacking *lapG* exhibits lots of red dots at the surface of the bacterial cells corresponding to RtxA protein embedded into the outer membrane as visualized in Fig. 5B. The confocal microscopy images clearly showed the presence of RtxA antibodies labelled in red close to the blue stained bacterial cells. Moreover, it is worth to note certain phenotypic characteristics such as aggregation, possibly due to the interaction between the RtxA proteins and neighboring cells, which may in turn cause clumping of the cells (Fig. 5A). The localization of RtxA at the surface of the cells was also revealed on many WT bacterial cells, dependent in that case on the activity/inactivity of LapG at the time of the experiment (Fig. 5B). Therefore, all these results confirmed that the LapD/LapG system controls RtxA localization (embedded or released) in *L. pneumophila* by blocking or promoting its N-terminus cleavage in the periplasmic space. Interestingly, amoebae and macrophage infection experiments using *L. pneumophila* ∆*lapG* strain that retains RtxA on its cell surface allowed us to visualize RtxA immunolabeled bacteria at the surface of eukaryotic cells as soon as 20 min post-infection, suggesting the efficiency of *L. pneumophila* in targeting its host cells (supporting information Figure S3).

### RtxA, a key role in the initial stage of virulence

**Fig. 6:**
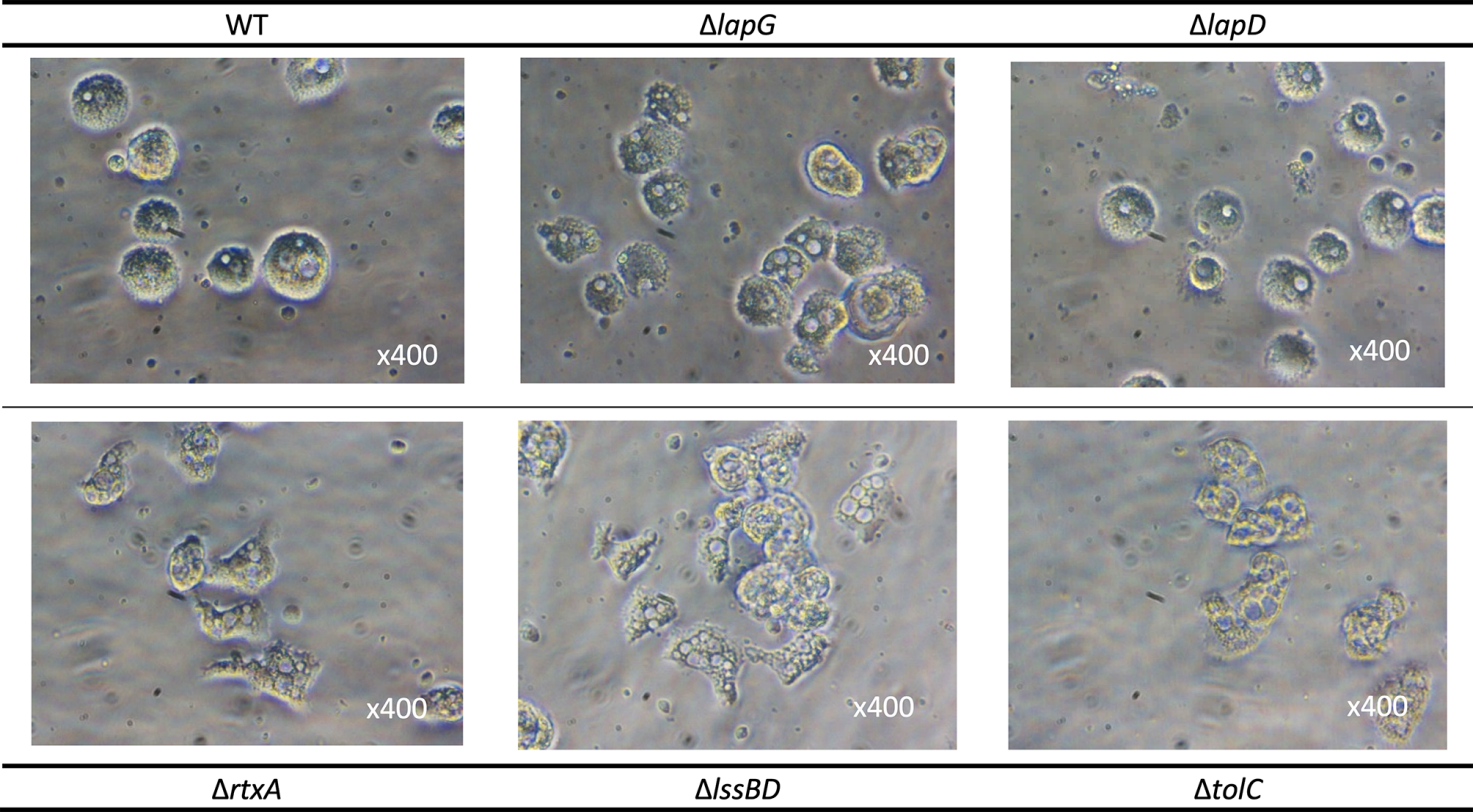
Importance of RtxA secretion and release systems in *L. pneumophila* virulence. *A. castellanii* were infected with *L. pneumophila* strains (WT, ΔlapG, Δ*lapD*. Δ*lssBD*, Δ*tolC* and Δ*rtxA*) *at* MOI 1 in a growth inhibitory medium. Light microscopy images were taken 3 days post infection. The round morphology of *A. castellanii* corresponds to stressed infected cells

The *rtxA* gene has been previously correlated to *Legionella* virulence, mainly adherence and entry into various host cells [12, 25, 26]. A mutant strain, Δ*dotA*, defective for Dot/Icm T4SS and hence intracellular replication was used as a negative control. All constructed mutants were tested for growth in AYE medium and no difference in growth capacity/fitness compared to wild-type strain was noticed (supporting information Figure S4 and Fuche et al., 2015). We then followed the progress of amoeba infection for the different strains using light microscopy. It is worth mentioning that all infection experiments made during this work followed a special protocol; *L. pneumophila* cells were added to host cells (amoebae or macrophages) without any centrifugation, hence avoiding forced contact and adhesion to host cells. Severity of the infection was based on amoebae mortality as well as morphology since they display a more round shape when infected [33], compared to the elongated morphology with possible pseudopods. We sought to recapitulate the insights gained above, regarding the regulated transport of RtxA to the bacterial cell surface, with the impact of RtxA on *L. pneumophila* virulence. Δ*rtxA* mutant, Δ*lssBD* and Δ*tolC* for the T1SS mutants, and Δ*lapD* or Δ*lapG* for the regulatory component of the RtxA localization were used to infect *Acanthamoeba castellanii*, as described above. Figure 6 images were captured three days after infection at MOI (multiplicity of infection) 1.

Two patterns can be observed regarding the severity of *A. castellanii* infection. Mutant strains for the T1SS, Δ*lssBD* and Δ*tolC* display lower virulence toward *A. castellanii*, a statement in agreement with our previous work with *L. pneumophila* Lens [25, 26]. Interestingly, Δ*lapD* infected cells were stressed in a manner similar to the WT infected cells whereas Δ*lapG* infected amoebae seemed to have a slight delay of infection (Fig. 6). However, monitoring the bacterial growth within amoebae during the infection showed no difference between WT, ∆*lapD* and ∆*lapG* strains (supporting information Figure S4). We can say that disrupting the T1SS responsible for RtxA secretion results in the lower virulence of these strains when compared to WT. It seems that manipulating the presence of RtxA on the cell surface (Δ*lapG*) versus being constantly released (Δ*lapD*) didn’t attenuate the virulence of *L. pneumophila* towards *A. castellanii* even if RtxA release seemed to be more favorable. Overexpressing *lapG* in WT strains also resulted in a small increase of fluorescence during amoebae infection which reinforces this hypothesis (supporting information Figure S4C). Complemented strains for the mutants were also constructed, e.g., *L. pneumophila* Δ*lapD*/pXDC50-*lapD* and Δ*lapG*/pXDC50-*lapG*. However, the axenic growth was altered in the case of Δ*lapD*/pXDC50-*lapD* strain with a start-up delay in AYE medium (supporting information Figure S4E). Moreover, the overexpression of *lapD* gene in the WT strain was deleterious in liquid media (supporting information Figure S4B). To better understand this phenotypic characteristic, we analyzed the transcriptional profile of the genes involved in RtxA secretion (T1SS), and release (*lapD*/*lapG*) at different physiological states: exponential, post-exponential (corresponding to the high infectious potential of *Legionella* with motility acquisition) and stationary phase (supporting information Figure S5). Interestingly, *lapD*/*lapG* genes were expressed at low level (~ 40 counts for *lapG* and ~ 100 counts for *lapD*) from the beginning of *Legionella* growth (exponential phase) and this expression decreased slowly during the post-exponential and stationary phase, corresponding to an early implementation of the complex in inner membrane and periplasm. A similar expression pattern was observed for *tolC*, mainly expressed during exponential phase (~ 2000 counts), in agreement with its pleiotropic role as a partner of efflux pumps complexes as well as in T1SS machinery. On the contrary, the inner membrane and periplasmic component of T1SS, *lssB* and *lssD*, were highly expressed during the post-exponential phase (from 3000 to 7000 counts) and corresponded to the beginning of RtxA expression which was still increased at stationary phase at a very high level (170,000 counts; supporting information Figure S5). Such a high production of RtxA is compatible with the release of RtxA that can be achieved when LapG cuts the N-terminal domain (108 AA). Thus, TolC now being free can interact with other substrate-engaged LssB/lssD and form a new T1SS complex. This RtxA hydrolysis is only possible if LapG is not sequestered by LapD in low c-di-GMP conditions. In the case of LapD overexpression, the constant “trapping” of LapG may result in a saturated T1SS linked to RtxA with the newly synthesized RtxA proteins retained in cytoplasm of bacteria cell with a toxic effect. However, the fact that ∆*lapG* mutants seemed viable without any toxic effect may reflect more complex organization and interactions between T1SS, RtxA and LapD/lapG components that still have to be elucidated.

In conclusion, transporting RtxA by the T1SS to the cell surface appears to be a crucial factor in *L. pneumophila* virulence efficiency, most likely in the early steps of infection such as host cell adhesion and/or recognition. We can also hypothesize that the presence of RtxA in the infection medium whether on the cell or released can play this role, independently of its location. However, this protein is not a limiting factor in *L. pneumophila* virulence as the infection can proceed without it albeit less efficiently.

### Protection against *L. pneumophila* infection of *A. Castellani* by anti-RtxA antibodies

**Fig. 7:**
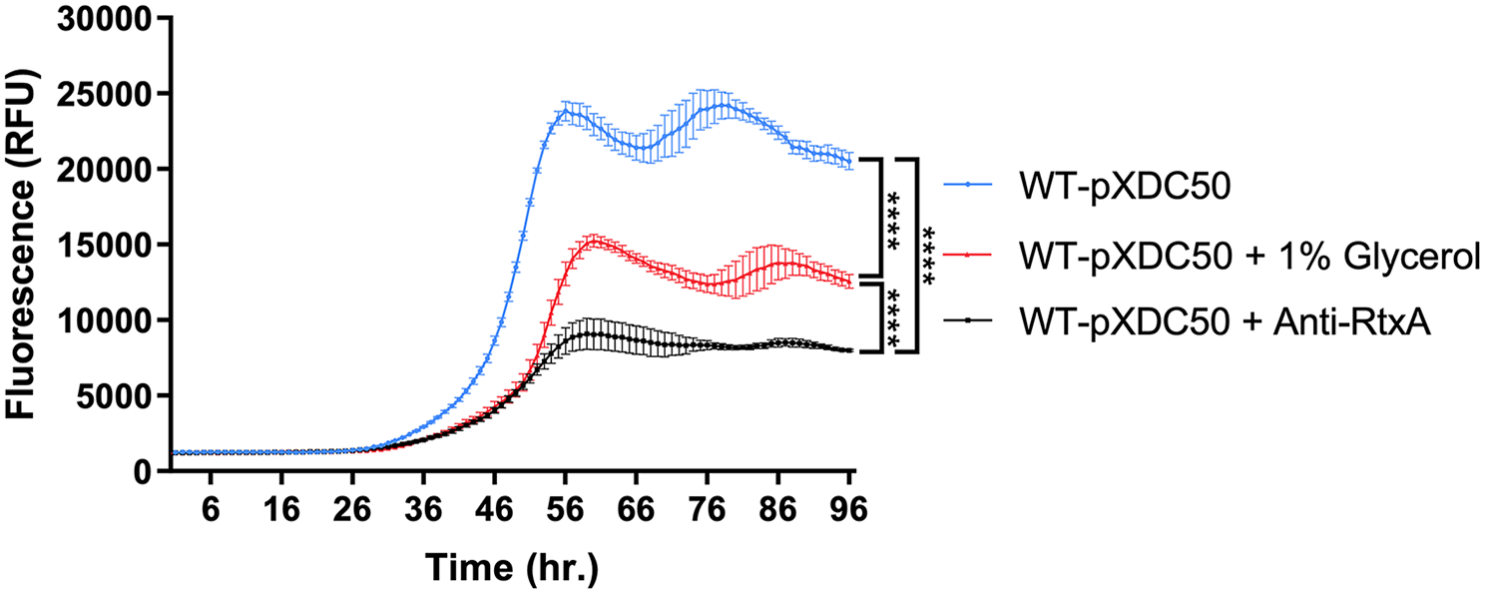
Anti-RtxA^COOH^ antibodies attenuate *L. pneumophila* infection of *A. castellanii. L. pneumophila* Paris wild type (WT) was used to infect *A. castellanii* cells at MOI 1. The curves represent variation in mCherry fluorescence versus time reflecting the number of bacteria. Results are displayed as mean fluorescence (3 replicates) ± one standard error of the mean. **** *P* < 0.0001 indicates that the means of our results are significantly different among each other. The significance was calculated by using a multiple comparisons test (Tukey’s test) to compare the mean of all time points in one infection experiment versus that of other infections at alpha 0.05

To further demonstrate the role of RtxA in *L. pneumophila* virulence, we monitored the effect of anti-RtxA antibodies on *A. castellani* infection. *A. castellanii* were infected by *L. pneumophila* in the presence of the purified *L. pneumophila* anti-RtxA^COOH^ antibodies described above. Since our antibodies were preserved in glycerol that might affect *L. pneumophila* virulence, appropriate controls were taken. Figure 7 shows that glycerol on its own is able to reduce the infection potential of *L. pneumophila* by approximately 36% even at 1% glycerol concentration.

However, the curve representing an infection in presence of 37.4 µg ml^− 1^ Anti-RtxA^COOH^ (containing an equivalent of 1% glycerol) was able to further decrease the peak fluorescence and consequently infection efficiency by approximately 62%. Taking into consideration that significance analysis confirmed the significance of our results at *P* < 0.0001, this suggests that anti-RtxA antibodies are able to hinder the infection process by binding and possibly disrupting RtxA role in amoeba infection.

### RtxA does not play a key role in the targeting of amoeba by *L. Pneumophila*

**Fig. 8:**
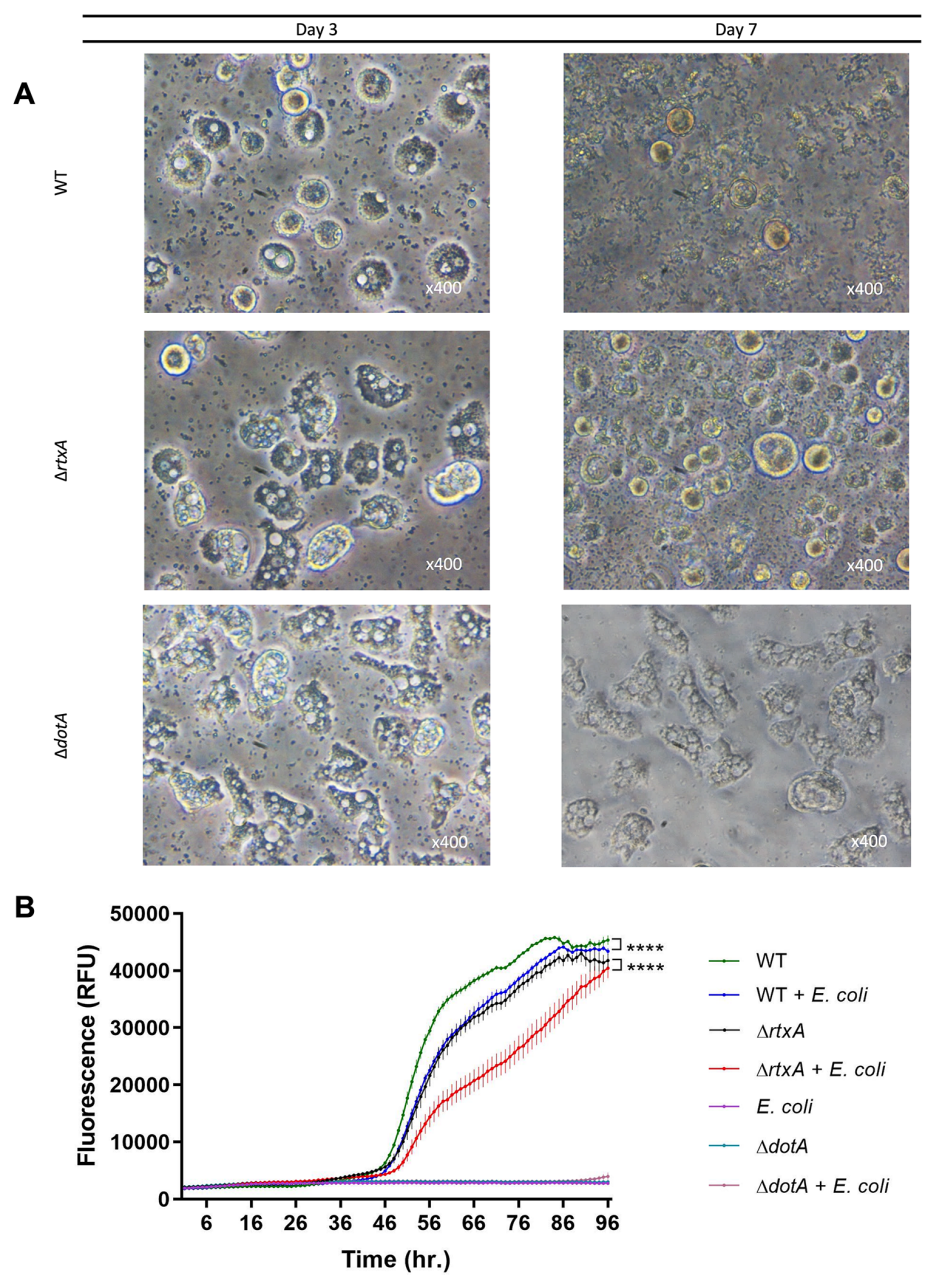
*L. pneumophila* may target *A. castellanii* during infection. **(A)** Infection of *A. castellanii* cells with different *L. pneumophila* strains (WT, Δ*rtxA*, Δ*dotA*) at MOI 1 in a growth inhibitory medium in the presence of 100-fold *E. coli* cells (MG1655) as a source of nutrition for amoebae. Observations were made after 3 and 7 days. Round morphology of *A. castellanii* corresponds to stressed infected cells. **(B)***L. pneumophila* Paris wild type (WT) and ∆*rtxA* mutant harboring pXDC50 plasmid were used to infect *A. castellanii* cells at MOI 1 in absence or presence of *E. coli* cells (100x more). The curves represent variation in mCherry fluorescence versus time reflecting the number of bacteria. Results are displayed as mean fluorescence (3 replicates) ± one standard error of the mean. **** *P* < 0.0001 indicates that the means of our results are significantly different among each other. The significance was calculated by using a multiple comparisons test (Tukey’s test) to compare the mean of all time points in one infection experiment versus that of other infections at alpha 0.05

In order to place the insights acquired here in an ecological context of interaction between *L. pneumophila* and its protozoan host, we assessed the role of RtxA in *L. pneumophila* virulence when the bacteria are in competition with other bacterial prey of amoebae. Indeed, phagotrophic protists including amoebae can be very selective consumers that recognize prey organisms [34]. Given the role of RtxA in the early steps of amoeba infection, we addressed the question whether RtxA could participate in the initial interaction/recognition/targeting between *L. pneumophila* and its environmental host. We thus investigated the effects of the presence of alternative prey cells of amoebae on the infection potential of *L. pneumophila*. We carried out *A. castellanii* infection with *L. pneumophila* WT, Δ*dotA* and Δ*rtxA* at MOI 1 in the presence of 100-fold excess cells of *E. coli* MG1655 (known prey of amoeba) in the infection medium. Figure 8A shows that after three days, *A. castellanii* cells infected with WT *Legionella* display significant stress, similar to a normal infection without *E. coli*, as observed above in Fig. 6.

Moreover, as expected, the ∆*rtxA* mutants displayed lower virulence than WT after three and seven days compared to WT (Fig. 8A and supporting information Figure S6). However, a quantitative assay using *L. pneumophila* strains expressing mCherry has shown that the infection cycle was modified in the presence of competitor *E. coli* cells (Fig. 8B). Although there is no clear delay in the onset of *L. pneumophila* WT growth within the amoebae, the fluorescence level is lowered at each point in the kinetics. That might be the result of a lower number of amoebae cells infected, thus lowering the fluorescence level as the total *Legionella* cells number replicating in the whole population of amoebae was decreased. This attenuation of infection potential was expected to be more emphasized in the presence of an alternative nutrient source for amoebae. In other words, the addition of an alternative nutrient source did not drastically alter the infection process. The attenuating effect of this competitor on infection was greater for ∆*rtxA* strain and might be the consequence of the slower capacity of this defective mutant to enter amoebae, thus giving advantage to phagocytosis of *E. coli* which might compensate *Legionella* entry. Keeping in mind that we did not force any contact between amoebae and *L. pneumophila* or *E. coli*, we can assume that *L. pneumophila* may actively target its host cell rather than simply being engulfed and consumed by phagocytic cells, even if the presence of a competitor in high excess (100x) may reduce the total of amoebae cells infected.

## Discussion

Bacterial surface-associated virulence factors are particularly important for adhesion and possibly evasion of the host cell responses [35]. One such class of membrane associated virulence factors include RTX proteins produced by a variety of Gram-negative bacteria. RTX proteins have diverse functions and range from being cytotoxins, hemolysins, proteases, lipases, and adhesins [36].

In this work, we show that *L. pneumophila* periplasmic protease LapG effectively cleaves *in-vitro* RtxA in its N terminal region. Moreover, by sequencing the first 7 amino acids of the cleaved fragment, we identify the exact cleavage site between amino acids 108 and 109. Previous research regarding *L. pneumophila* LapG protein revealed it was capable of cleaving the *P. fluorescens* LapA^Ntern^ albeit less efficiently than *P. fluorescens* native LapG protein [27]. Here, we demonstrate that *L. pneumophila* RtxA is indeed an endogenous substrate for LapG and the few amino acid substitutions surrounding the cleavage site of LapA compared to RtxA may explain the difference in cleavage efficacy observed by Chatterjee et al. We also assessed the effect of RtxA cleavage on its localization on the bacterial cell surface. Using *L. pneumophila* mutants and antibodies specific to the RtxA^Cterm^, we established that in Δ*lapD* mutant strains, no RtxA was detected at the surface of the bacterial envelope. Similar to the findings regarding *P. fluorescens*, we propose that LapD negatively controls, likely by sequestering LapG at the inner membrane, the LapG protease activity; in the absence of LapD, LapG is thus able to continuously cleave RtxA and release it from the cell surface. At the opposite, the absence of LapG protease activity in the Δ*lapG* mutant strain results in a significant presence of RtxA on the bacterial surface. Notably, the fixed localization of RtxA on the bacterial surface is associated with bacterial aggregation compared to WT and other mutant strains, which may be due to RtxA intermolecular interactions that result in the interaction of bacteria with each other [14]. Together, these data suggest that LapD has regulatory potential over LapG protease activity and that RtxA^Nterm^ cleavage results in its release from the cell surface, as already proposed in the LapA-LapG/LapD *Pseudomonas* model [14].

Looking for LapD/LapG proteins among translated bacterial genomes, we clearly identified homologous proteins in most *Legionella* species, and on a wider scale, in many gamma-proteobacteria species. This rules out the previous observation by Qin et al. [10] that restricted the T1SS-RtxA system to *L. pneumophila* species and reinforces its widespread presence in *Legionella* genus [30, 37], however in a less extended manner in non-*Legionella* species (around 25% considering co-occuring genes; Fig. 4B). We confirm here that deletion of *rtxA* definitely affects *L. pneumophila* virulence as the infection of amoebae was less severe in Δ*rtxA* genetic background compared to the wild-type, at least occurring after a delay. However, deletion of *lapG* and *lapD* genes did not result in clear virulence reduction in our conditions, which suggests that the localization of RtxA at the bacterial surface or released in the infection medium does not significantly impact the function of RtxA in *L. pneumophila* virulence towards amoebae. Our previous research already identified RtxA-dependent pore forming activity of *Legionella pneumophila* on eukaryotic cells [22] which could facilitate the entry of *Legionella* even if the protein is released from the surface.

This RtxA enhancing role during the early steps of the infection process was even clearer in the presence of a competitor (*L. pneumophila* with 100-fold more *E. coli*) as its absence resulted in the decreased number of infected amoebae cells. However, the absence of a delay in the onset of *L. pneumophila* WT infection process leads us to conclude that (i) *L. pneumophila* does not exclusively act as prey for environmental phagocytic cells, but likely can actively target its host cell, and (ii) this “targeting” potential of *Legionella* does not clearly depend on RtxA protein, which most likely plays a role in the next step, to promote bacterial entry into the host cell.

Using anti-RtxA antibodies specific to the C-terminal region in immunofluorescence imagery, we revealed at 20 min post-infection, fluorescent spots corresponding to the presence of RtxA at the surface of the host cells (amoebae and macrophages; supporting information Figure S3). This suggests that RtxA could adhere on eukaryotic cell surface probably with its adhesion motifs in C terminal and central repeat region [22]. Moreover, addition of anti-RtxA^COOH^ antibodies can affect the infection potential of *L. pneumophila* towards amoebae. In the presence of 37.4 µg ml^− 1^ of antibodies, there was a 62% reduction in virulence but glycerol accounts for around 36% of this effect as seen in the glycerol control. Interestingly, previous research reported the use of a C-terminal region of *Vibrio vulnificus* multifunctional auto-processing RTX protein (MARTX) called RtxA1 which is implicated in virulence to immunize mice, where this resulted in significant protection against lethal challenge with *V. vulnificus* [38].

In addition, preliminary results of co-immunoprecipitation of RtxA with anti-RtxA^COOH^ and anti-RtxA^NH2^ antibody from infected macrophage, analysed by mass spectrometry, provided prospective cellular targets of RtxA, among which the protein disulfide isomerase (PDI) P4HB (supporting information Figure S7). Interestingly, structurally intact PDI (with 95% of identity with P4HB) at the surface of the host cell is required for host cell adhesion and the reducing enzymatic activity of PDI is necessary for entry of several *Chlamydia* species [39, 40]. Importantly, there is also evidence that PDI regulates the activity and clustering of integrins [41] where several RTX toxins (HlyA, LtxA) have been described to interact with β2 integrins. Integrins are cell surface receptors which mediate multiple processes, including adhesion to other cells and the extracellular matrix [42]. Thus, RtxA may also stimulate adhesion of *L. pneumophila* strains to the host cell surface through the action of PDI on these receptors.

Notably, the RTX surface proteins that undergo a regulated release process, are associated with biofilm formation. We also detected cellular aggregation in mutant strains unable to release RtxA in the extracellular medium, which might hint at a role of bacteria-associated RtxA in biofilm formation as well. *L. pneumophila* LapD is a central module in the regulation process of the switch between embedded/released RtxA protein, similar to its homolog in *P. fluorescens* [14]. Further research is needed to unravel possible partners of LapD that allow such process to be tightly controlled in conjunction with the life cycle of *Legionella pneumophila*, specifically in biofilm and during infection. This raises the question of the primary role of RtxA in *Legionella*. As in many other bacterial species, it sounds reasonable to propose a major function in biofilm formation, promoting cells aggregation when RtxA is embedded in the outer membrane, facilitating biofilm dispersion in case of RtxA release. The RtxA enhancing role in the infection process may be the result of its action on host cells (i.e., PDI interaction or pore-forming implication), and independently of its status (embedded or released) as we showed in this work.

## Methods

### Strains and growth conditions

*Legionella* and *E. coli* strains used in this study are listed in Table 1.

**Table 1:** Bacterial strains and eukaryotic cells used in this study

Strain	Genotype	Source/Reference
XL1-Blue	*recA1 endA1 gyrA96 thi-1 hsdR17 supE44 relA1 lac* [F ´*proAB lacI*q*Z*Δ*M15* Tn*10* (Tetr)]	Stratagene
DH5-Alpha	F^–^*Φ80lacZΔM15* Δ*(lacZYA-argF) U169 recA1 endA1hsdR17 (rK–, mK+) phoA supE44 λ– thi-1 gyrA96 relA*	Invitrogen
BL21	F^–^*omp*T *hsd*S_B_(r_B_^–^, m_B_^–^) *gal dcm*	
Bl21/pREP4-*groESL*	F^–^*omp*T *hsd*S_B_(r_B_^–^, m_B_^–^) *gal dcm* carrying the plasmid pREP4-*groESL* (coding for LacI, GroES and GroEL)	Stratagene
***Legionella pneumophila***		
Paris	*L. pneumophila* serogroup 1, Paris strain	[43]
Paris Δ*dotA*	Δ*lpp2740*	This study
Paris Δ*tolC*	Δ*lpp*0889	This study
Paris Δ*lapG*	Δ*lpp*0890	This study
Paris Δ*lapD*	Δ*lpp*0891	This study
Paris Δ*lssBD*	Δ*lpp*1473, Δ*lpp*1474	This study
Paris Δ*rtxA*	Δ*lpp0699*	This study
**Eukaryotic cells**	**Phenotype**	**Reference**
*Acanthamoeba castellanii*	Environmental isolate	[44]

Regarding *L. pneumophila*, the Paris strain was used in the hereby presented work, it was grown on buffered charcoal yeast extract (BCYE) agar or in liquid BYE medium. Cultures were grown at 30–37 °C depending on experiment. Isopropyl β-D-1-thiogalactopyranoside (IPTG; 0.5 mM), kanamycin (15 µg ml^− 1^) or chloramphenicol (5 µg ml^− 1^) were added when appropriate. *E. coli* strains were grown in lysogeny broth (LB) with rotation or agar at 37 °C unless mentioned otherwise. Appropriate antibiotics were added when necessary, according to the following concentrations; kanamycin (50 µg ml^− 1^) and/or ampicillin (100 µg ml^− 1^). Axenic *Acanthamoeba castellanii* cells were grown on proteose-yeast extract-glucose (PYG) medium at 30 °C and split once a week (mechanically detached by tapping the flask and reseeded in fresh PYG at 1/10 dilution), appropriate antibiotics were supplemented.

### Plasmid construction

DNA constructs used in this study were made by using *E. coli* BL21 (pET30a and PQE30 constructs) or DH5-α (pXDC50 constructs) as the host. Plasmids used and constructed in this study are described in Table 2.

**Table 2:** Plasmids used in this study

	Base Plasmids	
Plasmid	Characteristics	Source/Reference
pXDC50	Utilizable plasmid in *Legionella*, mCherry expression (Ptac promoter), *lacIq*, CmR	[45]
pET-30a(+)	Bacterial expression plasmid, T7 promoter, 6xHis tag – N&C^term^, KanR	Sigma-Aldrich
pQE30	Bacterial expression plasmid, T5 promoter, 6xHis tag – N^term^, AmpR	QIAGEN
pGEX-6p-3	Bacterial expression plasmid, Ptac promoter, GST tag – N^term^, AmpR, PreScission cleavage site	Sigma-Aldrich
**Plasmids for mCherry expression and deletion complementation in** ***L. pneumophila***		
pXDC50-*lapG*	*lapG* gene of Paris strain (*lpp*0890) cloned by XbaI/SphI in pXDC50 (under Ptac control); *lacIq*, CmR	This study
pXDC50-*lapD*	*lapD* gene of Paris strain (*lpp*0891) cloned by XbaI/SphI in pXDC50 (under Ptac control); *lacIq*, CmR	This study
**Plasmids for overproduction of RtxA and LapG in** ***E. coli***		
pQE30-LapG	*lapG* gene of Paris strain (*lpp*0890 without signal sequence) (192 a.a.) cloned by BamHI/PstI in pQE30 (T5 promoter), KanR	This study
pET30a-RtxA^NH2^	N-term of RtxA (492 a.a.) belonging to gene *lpp*0699 cloned by NdeI/SalI in pET30 (T7 promoter); KanR	This study
pGEX6p3-RtxA^COOH^	C-term of RtxA (a.a. 6490–6764) belonging to gene *lpp*0699 cloned by EcoNI/EcoRI downstream a GST tag in pGEX6p3 (Ptac promoter), AmpR	This study

*L. pneumophila* Paris strain genomic DNA was used as template for PCR production of desired inserts. pXDC50 plasmid [45] was obtained from Xavier Charpentier. The appropriate plasmids were inserted into *L. pneumophila* by electroporation (2400 V, 400 Ω, 25 µF). The recombinant plasmids were verified by PCR and enzymatic digestion.

### Gene deletion in *L. Pneumophila*

Gene-specific deletions in *L. pneumophila* were carried out using the homologous recombination method [46]. Mutant strains were derived from *L. pneumophila* wild type (WT) Paris strain. Briefly, this two-step process relies on the natural competence of *L. pneumophila* and results in clean scar-free mutants. In our work, the 2 kb regions flanking the gene to be deleted were amplified by PCR. In the first step, a resistance/suicide inducible cassette which is in this case kanamycin/MazF, is inserted between the flanking regions by double joint PCR. This constructed cassette introduced into *L. pneumophila* competent cells will replace the gene of interest and will allow for primary selection on kanamycin supplemented media. Successful colonies are kanamycin resistant and IPTG sensitive (Kan^r^ IPTG^s^). The cassette integration was confirmed by PCR. In the second step, the gene flanking regions were joined by PCR and used for natural transformation of the previously obtained “first step mutants”. The aim is to replace the Kan/MazF cassette which will produce clean gene deletions. Colonies corresponding to deleted mutants are Kan^s^ IPTG^r^ and DNA region deletions were also confirmed by PCR.

### In vitro LapG cleavage analysis

RtxA^Nterm^ (nucleotides 1-1490 from *rtxA* lpp0699) was cloned into a pET-30 plasmid upstream a 6xHis Tag using NdeI/SalI restriction sites to insert the DNA fragment. The constructed plasmid allowed for production of an N-terminal fragment of 505 amino acids including 6 histidine with a molecular mass of 53.2 kDa. In brief, the constructed plasmid was transformed into *E. coli* strain BL21 by electroporation. The recombinant protein was produced by a 2-hour induction with 0.1mM Isopropyl β-D-1-thiogalactopyranoside (IPTG) of an exponential culture that reached an OD_600nm_ of 0.5. Cells were collected and lysed using a French pressure cell (14,000 Psi). The recombinant protein was purified using Talon^®^ metal affinity resin (Takara Bio USA Inc) and eluted in the provided elution buffer: 50 mM sodium phosphate, 300 mM NaCl, 150 mM imidazole, pH 7.4. Similarly, *L. pneumophila* LapG was produced in *E. coli* BL21 by cloning of nucleotides 169–735 (lpp0890) using BamHI/PstI restriction sites to insert the DNA fragment in a pQE-30 plasmid downstream a 6xHis tag. The recombinant protein (22.5 kDa) was purified as described earlier. To assess the protease activity of *L. pneumophila* LapG on RtxA, the purified proteins (in elution buffer) were co-incubated for 3 h at 37 °C in the presence of 40 mM CaCl_2_ and 80 mM MgCl_2_. The hydrolyzed fragments were observed using SDS-PAGE. A second hydrolysis mix was separated by SDS-PAGE and transferred onto a PVDF membrane by electroblotting. Coloring the membrane with Ponceau red allowed the excision of the cleaved RtxA band that has been rinsed in water for further processing: the first 7 amino acids of the cleaved RtxA^Nterm^ (C fragment) were sequenced by Edman degradation.

### Production of RtxA N- and C-terminus polyclonal antibodies

The desired fragments were designed, expressed and purified to obtain a protein sample for antibody production. Briefly, the RtxA^NH2^ gene portion (nucleotides 14-1490 from *rtxA* lpp0699) was cloned into a pET-30 plasmid upstream a 6xHis Tag; the RtxA^COOH^ gene portion (nucleotides 19,482 − 20,309 from *rtxA* lpp0699) was cloned into a pGEX-6P-3 plasmid downstream a glutathione S-transferase (GST) tag followed by an HRV 3 C site for cleavage by a PreScission protease. The constructed plasmids allowed for production of N and C-termini fragments of 492 and 278 amino acids respectively. In brief, the constructed plasmids were transformed into BL21 and XL1-Blue *E. coli* strains respectively. Recombinant proteins were produced using an overnight culture followed by a 2-hour induction with 0.1mM IPTG. Cells were collected and broken using a French pressure cell (14,000 Psi). The recombinant proteins were purified using Talon^®^ metal affinity resin (Takara Bio) for his-tagged proteins, and GST affinity resin (Protino^®^ Glutathione Agarose 4B; Macherey-Nagel, Germany) for GST tagged proteins. The purity and size of the expressed proteins were assessed by SDS-PAGE and quantified using a modified Bradford assay (Roti^®^-Quant; Carl Roth, Germany). Purified polyclonal antibodies were produced commercially (Covalab, France) by immunization of rabbits with purified RtxA N-terminus or C-terminus (elution samples), then passing the antisera through columns charged with our recombinant proteins followed by elution. Specificities of the antibodies were confirmed by western blots of whole cell lysates.

### *Intracellular growth of L. pneumophila*

*A. castellanii* cells were washed in modified proteose-yeast extract medium lacking peptone, yeast extract and glucose (PYspecial) then seeded into a 24-well tissue culture treated microplate (Greiner CELLSTAR^®^, Germany) at 1 × 10^5^ cells/well and left to adhere for 2 h at 30 °C. Amoebae were infected at a multiplicity of infection (MOI) of 0.1 with bacterial suspensions in PYspecial made by dilution of late-stationary phase cultures of *L. pneumophila* strains. It is important to note that many protocols require centrifugation of the microplates after inoculation with bacteria to force contact between *L. pneumophila* and its host; we skipped this step to avoid forcing the infection process by *Legionella*. Plates were then incubated at 30 °C and microscopic observations were performed daily to follow the progress of infection. As a competition assay, we repeated the same infection procedure described above at MOI 1 but with supplementing the infection medium with 1 × 10^7^*E. coli* MG-1655/well (i.e., 100x *Legionella*) as an alternative source of nutrients for *A. castellanii*.

### Protection against infection using Anti-RtxA antibodies

*A. castellanii* cells were washed then seeded into a 96 well microplate (Greiner CELLSTAR^®^, Germany) at 1 × 10^5^ and left to adhere for 2 h at 30 °C. Amoebae were infected at a MOI of 1 with bacterial suspensions made by dilution of late-stationary phase cultures of *L. pneumophila* strains. The infection medium used (proteose-yeast extract) is bacteriostatic for *Legionella*, only allowing intracellular growth. The *Legionella* strains used in this experiment were transformed with a pXDC50 plasmid expressing mCherry red fluorescent protein. The infection medium in both cases was supplied with chloramphenicol (5 µg ml^− 1^) and IPTG (1 mM). Before inoculation, we added different concentrations of purified anti-RtxA^COOH^ to the bacterial suspension. Appropriate controls were performed including various concentrations of glycerol since it was used to preserve our antibodies. To measure fluorescence, the plates were incubated for 3 days in a Tecan Infinite^®^ F200 pro at 30 °C.

### Immunofluorescence microscopy

To visualize RtxA proteins on the surface of *L. pneumophila*, we modified a procedure based on a previous protocol [47]. The first step was preparing glass coverslips by cleaning them in Ethanol/HCl solution (1 M) for 1 h then coating with poly-L-lysine (Sigma P8920). The following steps were carried out at room temperature (RT). *Legionella* cells were normalized to O.D_600nm_ 1.0 in Dulbecco’s phosphate-buffered saline (DPBS; Gibco 1490-094) and 300 µl of this suspension were spread on the previously prepared coverslips, then left to adhere for 30 min. The excess suspension was then gently aspirated and replaced with 3.7% formaldehyde (Acros Organics 119,690,010) and left 30 min to fix the cells. After rinsing with DPBS, non-specific sites were blocked by 3% bovine serum albumin (BSA) solution in DPBS for 30 min. After blocking, the slides were washed again with DPBS and 100 µl (1:10000–0.374 µg ml^− 1^ in DPBS) of primary antibody (rabbit anti-RtxA^Cterm^) were added to the coverslips followed by incubation for 1 h. After three washes with DPBS, 50 µl of fluorescence conjugated secondary antibody (Alexa Fluor^®^ 568 goat anti-rabbit, Invitrogen Inc. USA) were incubated with the coverslips for 1 h in the dark. The slips were then washed twice and mounted on glass slides with 20 µl mounting medium (Mowiol^®^+DAPCO) (Sigma-Aldrich, USA). Finally, cells were visualized using an inverted fluorescence microscope (Thermo Fisher EVOS FL AME4300, USA) using LED light cubes: AMEP 4650 EVOS™ (DAPI), AMEP4651 EVOS™ (GFP) and AMEP 4652 EVOS™ (RFP). The microscopy preparations were visualized through the coverslips using a plan fluorite x40/0.65 objective and the transmitted light images were done using Bright-field contrast. To achieve the confocal microscopy observations, the same samples preparations were done with 2 modifications: the secondary antibody was the Alexa Fluor^®^ 594 goat anti-rabbit (Invitrogen Inc. USA) and the bacteria cells were stained with DAPI prior mounting on glass slides. The preparations were visualized through the coverslips using an inverted confocal microscope Zeiss LSM 800 with a plan-apochromat 63x/1.40 oil M27 objective.

### Statistical analysis

Protection against *L. pneumophila* infection using anti-RtxA antibodies were performed in triplicate and the results were displayed as mean values ± one standard error of the mean. Differences in protection efficiencies were evaluated by ordinary two-way ANOVA for analysis of variance followed by Tukey’s multiple comparisons test of significance for means. These analyses were performed using GraphPad Prism version 8.4.0 for Windows, GraphPad Software, San Diego, California USA, www.graphpad.com. P value less than 0.05 were considered statistically significant.

### Sequence searches, alignments and Phylogenetic studies

Homologous protein sequences were searched on NCBI website (https://www.ncbi.nlm.nih.gov/) using Blastp software. Proteins of interest (one per species identified) were downloaded as FASTA files to perform further analysis. Alignment of RtxA cutting site regions was done using Jalview software using Clustal Omega software to align the protein sequences (version 2.10.5; [29]). Phylogenetic trees were inferred using maximum-likelihood with PhyML 3.0 software online pipeline (http://phylogeny.lirmm.fr; [31]).

To determine the presence or the absence of proteins in *Legionella* genomes (647 genomes), the protein sequences of interest of *L. pneumophila* were blasted (Blastp software) against our family-clustered proteins database [32].

### Electronic supplementary material

Below is the link to the electronic supplementary material.

Supplementary Material 1

## Data Availability

The datasets used and/or analysed during the current study are available from the corresponding author on reasonable request.
