# Supplementary material for: Legionella pneumophila cell surface RtxA release by LapD/LapG and its role in virulence
Source: BMC Microbiol. 2024 Jul 19;24:266. doi: 10.1186/s12866-024-03395-1 (PMC11264772; doi:10.1186/s12866-024-03395-1)
Supplement: Supplementary file 1 — Supplementary Material 1 [file 12866_2024_3395_MOESM1_ESM.pdf]

Supplementary Material

Legionella pneumophila cell surface RtxA release by LapD/LapG and its role in virulence

Hussein Kanaan, Annelise Chapalain, Ali Chokr, Patricia Doublet, Christophe Gilbert*

***Correspondence**: Christophe Gilbert, CIRI, Université Lyon 1, Bât. Rosalind Franklin, 50 avenue Tony Garnier, 69007 Lyon, France. Tel: (33) 4 37 28 74 82, christophe.gilbert.bio@univ-lyon1.fr

1. Supplementary Materials and Methods

a. *Strains and growth conditions*

Human U937 (ATCC® CRL-1593.2) cells were maintained at 37°C and 5% CO_2 in RPMI-1640 medium supplemented with 10% heat inactivated fetal bovine serum (FBS). Differentiation into macrophages was initiated by adding Phorbol 12-myristate 13-acetate (PMA) at a final concentration of 100 ng ml^{-1}.

b. *L. pneumophila* infection of phagocytic cells

U937 cells were seeded and then infected with Δ*lapG L. pneumophila* as previously described, however the infection was allowed to proceed for 15 minutes only. The infection was stopped and several washes PBS to remove non adherent cells. Then we added 250 µl of 4% paraformaldehyde (PFA) solution and left 30 it minutes to fix the cells. Finally, we washed again and added 250 µl PBS and the cells were visualized using an epifluorescence microscope (EVOS® FL; Thermo Fisher, USA).

c. *Immunofluorescence microscopy of L. pneumophila infection*

Infection experiments were carried out mainly in 96 well plates (Greiner CELLSTAR®, Germany). Regarding U937 cells, $1x10^5$ monocytes were seeded in each well and left to differentiate into mature macrophages for 2~3 days. Macrophages were infected at a MOI of 10 with bacterial suspensions made by dilution of late-stationary phase cultures of *L. pneumophila* strains; the infection medium used was RPMI-1640 + 10% FBS. The plate was then left for 20 minutes at 37°C. As for *A. castellanii*, $1x10^5$ cells/well were seeded and left overnight at 30°C, *L. pneumophila* were prepared as described before, but the infection medium in this case is the modified bacteriostatic proteose-yeast extract medium. After inoculation, the plate was left for 20 minutes at 30°C for the infection to proceed. The following steps were common for both cell types and were carried out at 30°C. The infection was stopped then we added 250 µl of 4% paraformaldehyde (PFA) solution and left 30 it minutes to fix the cells. After washing with phosphate-buffered saline (PBS pH 7.0), we added 250 µl DPBS + 0.1% glycine to reduce background fluorescence. Non-specific sites were blocked by DPBS + 3% bovine serum albumin (BSA) solution for 1 hour. The wells were washed again and 100 µl (1:10000 or 0.374 µg ml^{-1}) of primary antibody (rabbit anti-$RtxA^{COOH}$) were added and left to incubate for 1 hour. After several washes with DPBS, 50 µl of red fluorescence conjugated secondary antibody (Alexa Fluor® [568 for *A. castellanii* and 488 for U937 cells] goat anti-rabbit antibodies; Invitrogen Inc. USA) were added and the plates were left in the dark for 1 hour. Finally, we washed the wells twice with PBS and the cells were visualized using an epifluorescence microscope (EVOS® AME4300, Thermo Fisher, USA).

d. RNA Isolation and Depletion of rRNA and RNAseq

L. pneumophila Paris WT strain was grown at 37°C in AYE medium and harvested by centrifugation (5 min, 7000 rpm, 4 °C) at different growth phases: at the exponential phase (optical density of 1.5 at 600 nm (OD 600nm 1.5)), post-exponential phase (OD 600nm 4 and visual check of motility acquisition), and to the onset of stationary growth phase (OD 600nm 5). Total RNA from bacterial cultures was extracted according to a previously described procedure [44]. Briefly, pellets of 10^9 bacterial cells were lysed in 50µl of RNAsnap buffer (18 mM EDTA, 0.025% SDS, 95% formamide), and total RNAs were extracted using a tri-reagent solution (acid guanidinium thiocyanate–phenol–chloroform) and isopropanol-precipitated. After precipitation, we performed an additional step of RNA purification on silica-based columns (DirectZol kit, ZymoResearch) by following the manufacturer's recommendations. RNA sample purity and concentration were determined by spectrophotometric analysis on a NanoDrop 2000 UV-Vis spectrophotometer (Thermo). RNA sequencing was performed following ribosomal RNA depletion and cDNA library preparation on an NovaSeq platform (Illumina) with paired-end 150 bp (Genewiz-Azenta, Leipzig, Germany). After mapping sequence reads to the reference genome and extraction of gene hit counts, the comparison of gene expression between the defined groups of samples was performed using DESeq2.The BAM files were imported into IGV software (V2.15.2), and reads were aligned with the genome sequence of L. pneumophila Paris strain (NCBI accession number: NC_006368). We used IGV to visualize data as a graphical display to compare the transcriptomic data between *lssB*, *lssD*, *tolC*, *lapD*, *lapG* and *RtxA* obtained from the different experiments.

e. Cross-linking of infected cells

Infection of macrophages was carried out in the Petri dish used for their differentiation from monocytes. *L. pneumophila* strain Paris suspension was added to the plate at MOI 10 and the plate was then kept at 37°C for 30 min at CO_2 of 5 %.

Infected cells were centrifuged for 10 minutes at 25°C at 300 g. The pelleted cells were cross-linked with a 1% formaldehyde-stabilized solution and quenching was done with a solution of glycine 2,5 M. Cells were washed with DPBS 1X pH 7.4, pelleted and then suspended in 600 µL of IP Lysis/Wash Buffer (Pierce® Kit) for lysis with a Fast-Prep beader (MP Biochemicals).

f. Co-immunoprecipitation assays

Cell extracts from lysed infected macrophages were clarified using a Pierce® Crosslink Immunoprecipitation Kit column (ThermoFisher). Antibodies targeting the N-terminus or C-terminus of RtxA were obtained from Hussein Kanaan (PhD student in the team). The antibodies were coupled to the A/G protein resin from the Pierce® Kit and preserved in a solution containing 0.02% azide. Co-immunoprecipitation assays were performed after incubating the clarified cell lysates with one of both antibodies coupled to the resin. All was done according to the manufacturer's instructions.

2. Supplementary Figures

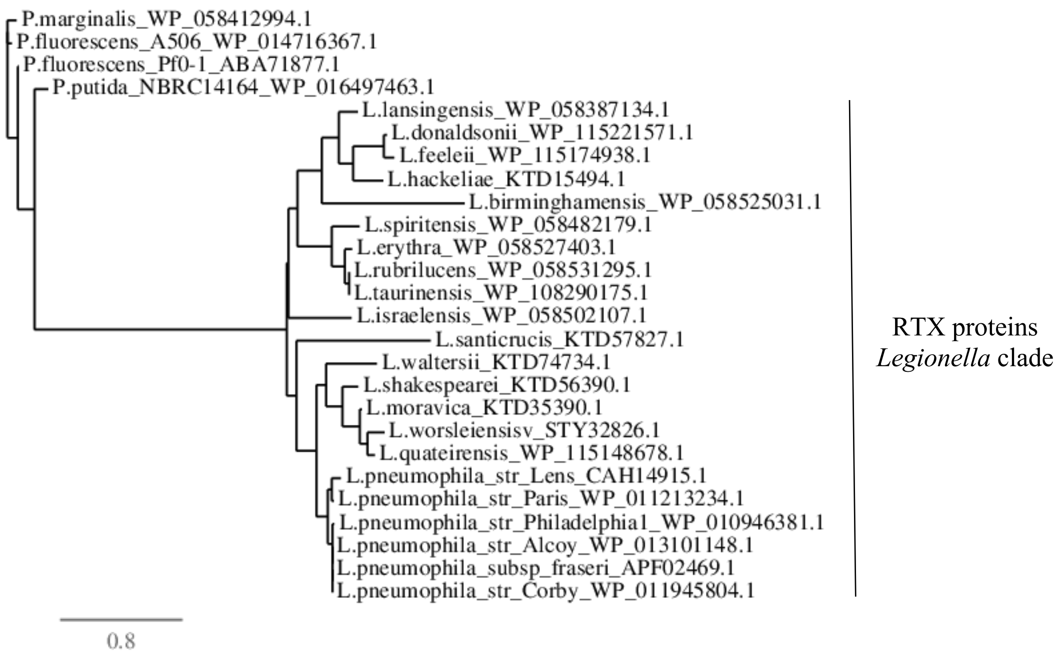

Figure S1: RTX family proteins phylogenetic tree inferred using maximum-likelihood (PhyML 3.0 software). The proteins reported in the tree correspond to RTX proteins shown on figure 2 alignment. The first N-terminal 210 aminoacids sequences of predicted proteins were used to perform the phylogeny. The branch length is proportional to the number of substitutions per site (scale at the bottom). L: *Legionella*; P: *Pseudomonas.*

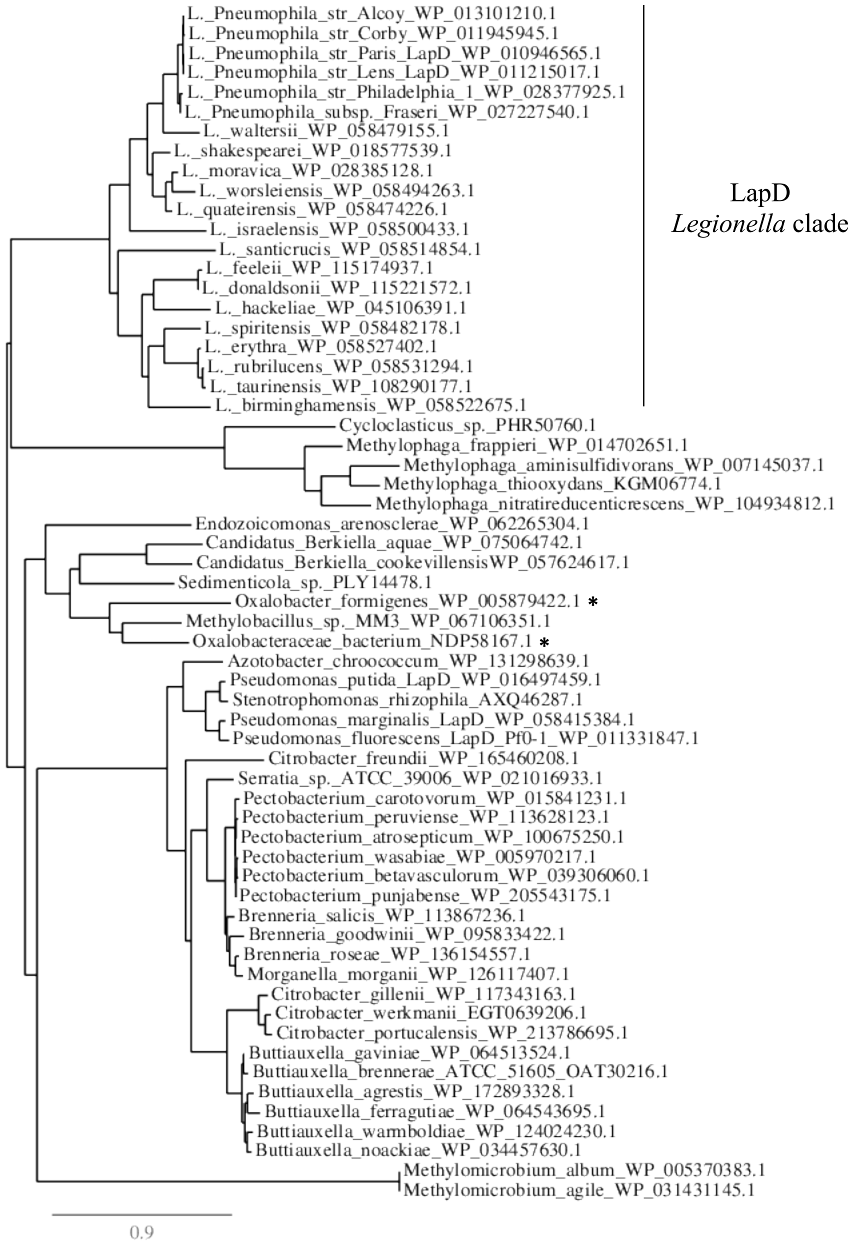

Figure S2: LapD family proteins phylogenetic tree inferred using maximum-likelihood (PhyML 3.0 software). The proteins reported in the tree were chosen for their high similarities with *Pseudomonas* LapD. Only one representative protein is conserved in each bacterial species except in *Legionella pneumophila*. All bacterial species are gammaproteobacteria except 2 pointed with an asterisk which are in betaproteobacteria class. The branch length is proportional to the number of substitutions per site (scale at the bottom). L: *Legionella.*

A. castellanii	U937 Macrophage

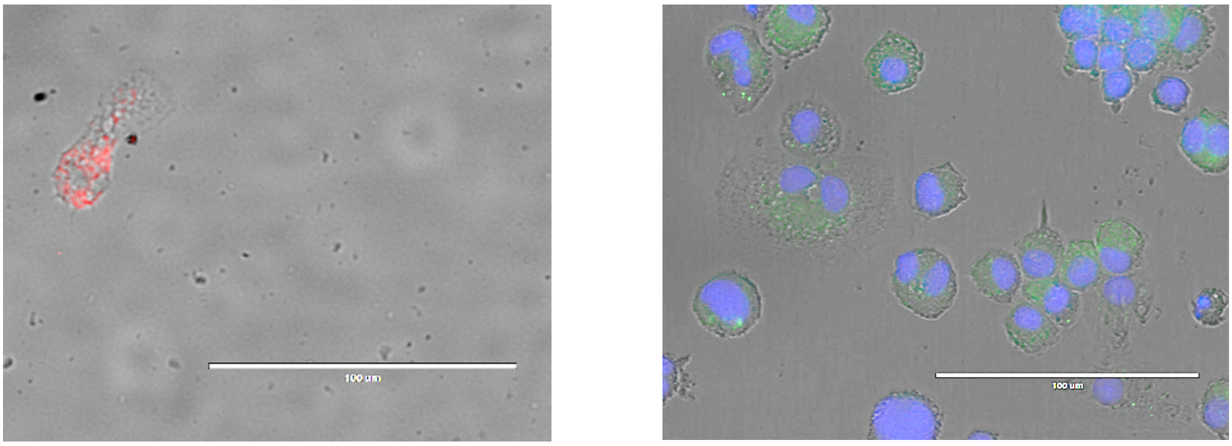

Figure S3: ***L. pneumophila*** **is detected on its host early during infection**

(Left) *A. castellanii* were infected with 3 days old Δ*lapG L. pneumophila* Paris strain, Anti-RtxA^{COOH} antibodies linked to a red fluorescence element (Alexa Fluor® 568 goat anti-rabbit), fluorescence is detected on the amoebae surface 20 minutes post infection. (Right) U937 macrophages were infected in a similar manner but using a green fluorescence element (Alexa Fluor® 488 goat anti-rabbit) and DAPI staining. Appropriate controls with no primary antibody were taken into account. Scale bar: 100 µm.

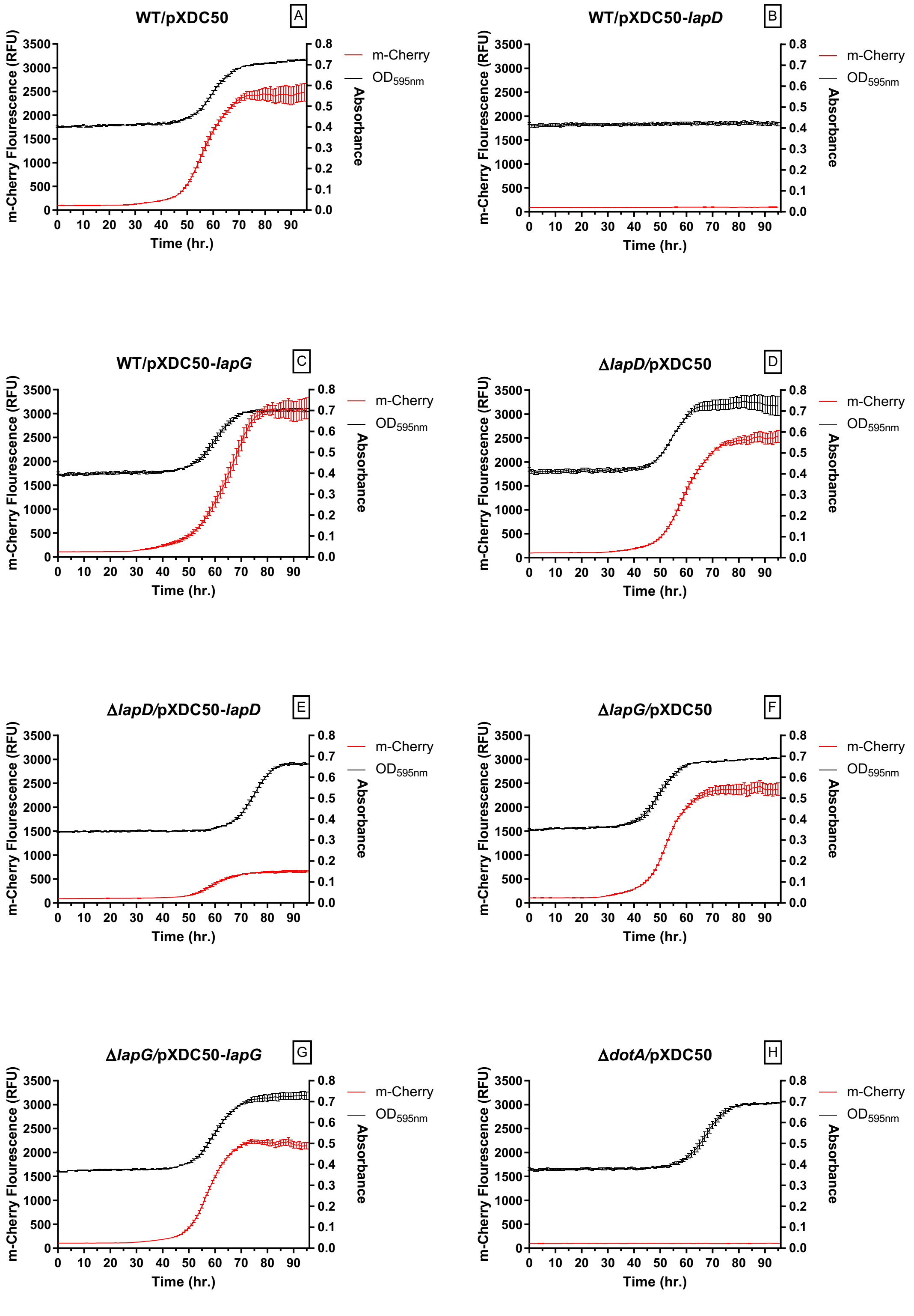

Figure S4: Growth of *L. pneumophila* Paris and derivatives strains and Infection of *A.castellanii* at MOI 1

L. pneumophila WT, mutants, and complementation strains were used to infect *A. castellanii* at MOI 1 for 4 days at 30°C in a multimode plate reader (TECAN infinite F200 pro). mCherry fluorescence represents *L. pneumophila* virulence in amoebae while OD_{595} represents its growth in AYE medium. An infection with a strain deficient in the type IV secretion system (Δ*dotA*) which is incapable of intracellular replication was used as a control (H).

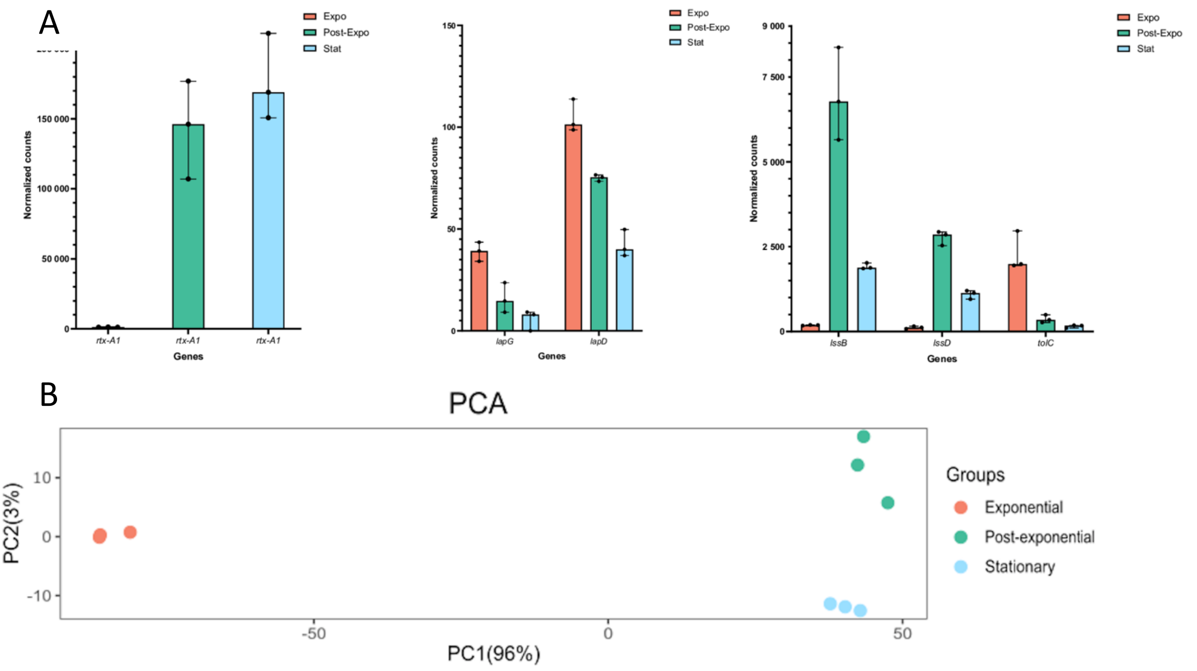

Figure S5: Transcriptomic analysis of essential genes involved RtxA secretion and release in *Legionella pneumophila* Paris.

Three cultures of *L. pneumophila* Paris WT were grown in liquid medium AYE (37°C). At the desired growth phases (exponential/OD 600 = 1 5, post-exponential/start of mobility acquisition /OD600 = 4, and stationary collected 2 hours after the post-exponential sample), samples were collected, and their RNA content was analyzed by RNAseq. (A) The graphs show normalized read counts of *lssB/lssD*/*tolC* (T1SS), *lapD*/*lapG* (RtxA release function) and *rtxA* mRNAs for each sample at the different growth phases (three repeats). (B) Principal Component Analysis (PCA) of the three repeats in each condition. The analysis clearly showed that the data corresponding to each condition are grouped and that each condition displayed a different profile of mRNAs count.

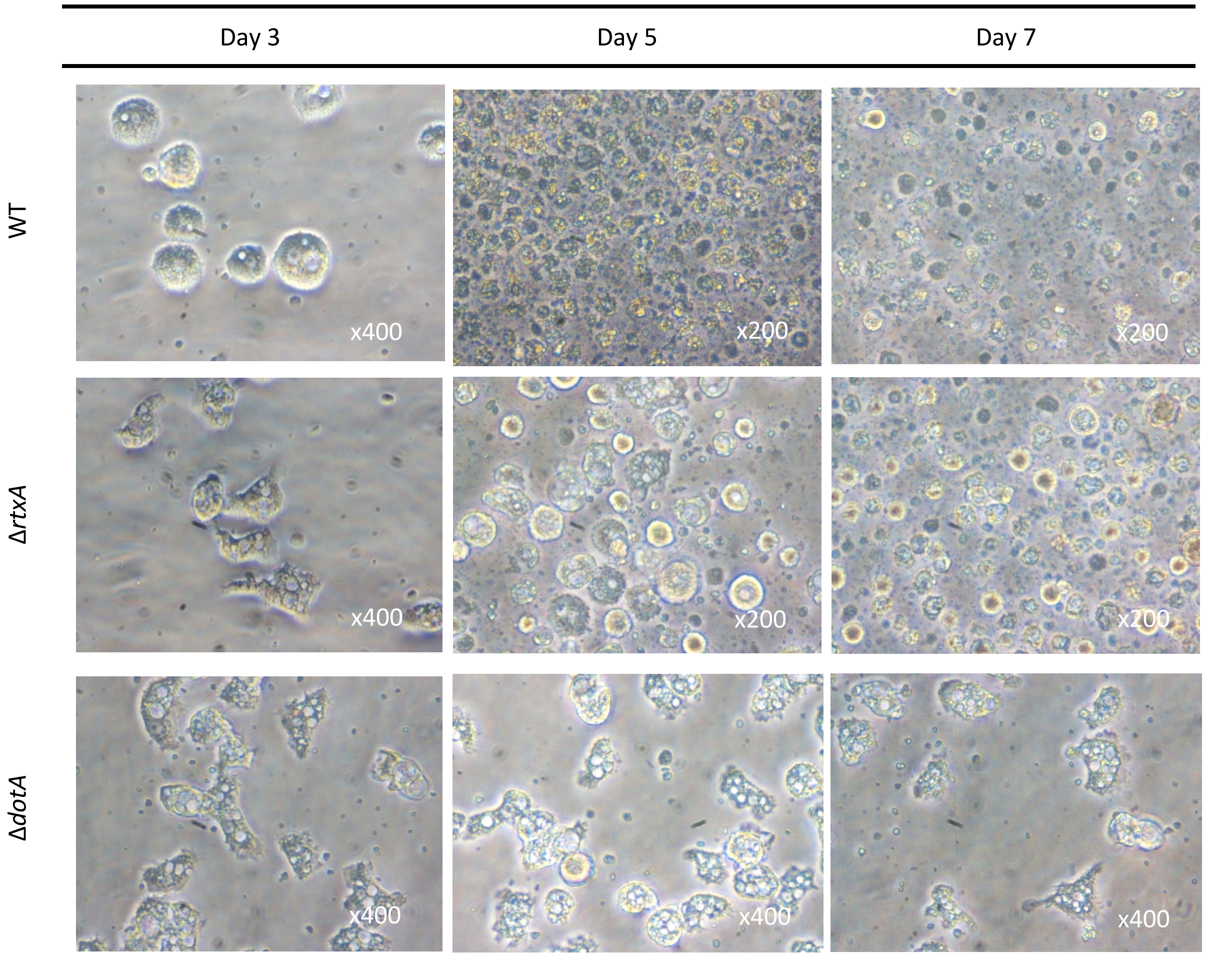

Figure S6: impact of *L. pneumophila* RtxA on the severity of *Acanthamoeba castellanii* infection

Three *L. pneumophila* strains were used for infection of *A. castellanii* at MOI 0.1 in a medium lacking growth requirements for both bacteria and its host. Light microscopy images were captured at 3 time points post infection. Round morphology of *A. castellanii* corresponds to stressed infected cells. An infection with a strain deficient in the type IV secretion system (Δ*dotA*) which is incapable of intracellular replication was used as a control.

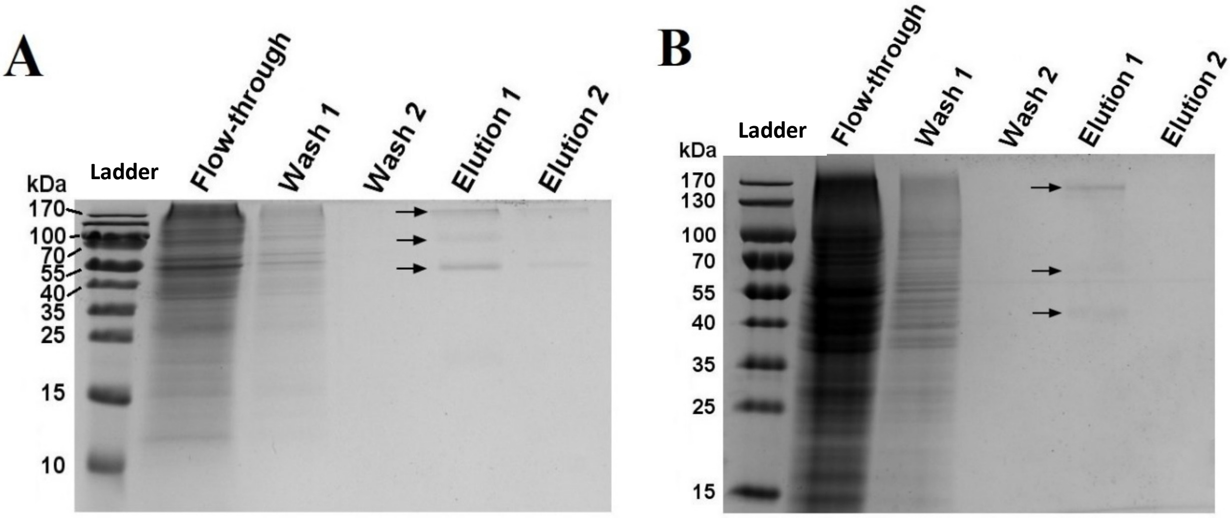

Figure S7: Co-immunoprecipitation (coIP) assays of macrophage lysates with anti-RtxA-antibodies

Samples were analysed by SDS-PAGE (**A)** CoIP of infected macrophage lysates with an anti-RtxA^{COOH} antibody (30 min. infection). (**B)** CoIP of infected macrophage lysates with an anti-RtxA^{NH2} antibody (30 min. infection). Gels were stained with Coomassie blue. In both cases, the arrows point out the three proteins bands clearly identified in elution fractions that were not present in control CoIP using uninfected macrophages.

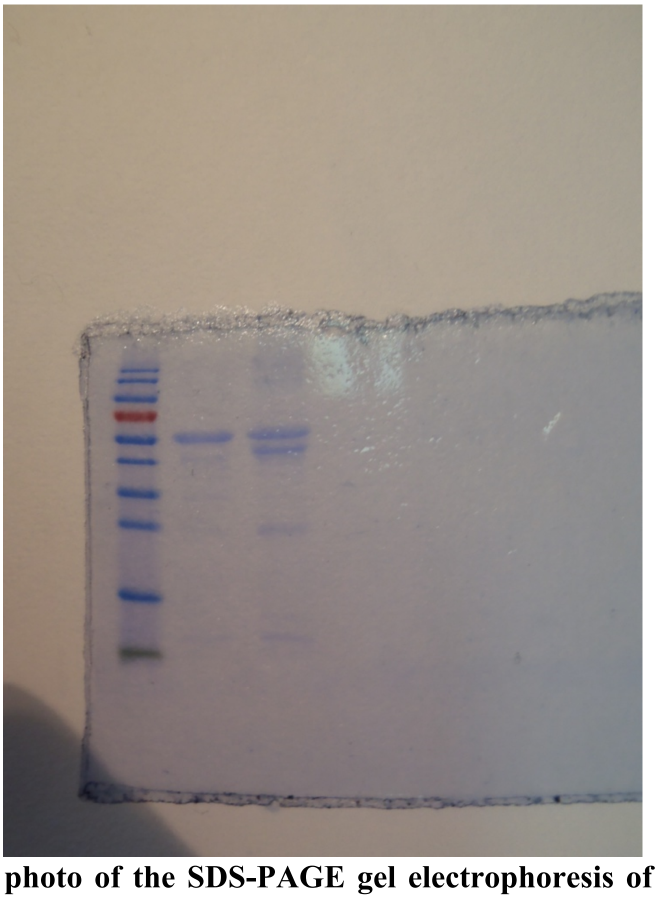

Figure S8: Original photo of the SDS-PAGE gel electrophoresis of figure 1 (RtxA^{Nterm} incubated with LapG protease).
